# Data ownership in genomic research consortia

**DOI:** 10.1093/jlb/lsae024

**Published:** 2024-12-06

**Authors:** Jane Nielsen, Dianne Nicol

**Affiliations:** Faculty of Law, University of Tasmania, Private Bag 89, Hobart, Tasmania 7001, Australia; Faculty of Law, University of Tasmania, Private Bag 89, Hobart, Tasmania 7001, Australia

**Keywords:** consortia, data, genomics, intellectual property, privacy, property

## Abstract

Discourse around ownership of genomic sequence data has proliferated over recent years. There are likely to be few people who don’t feel a degree of connectedness to their genomic data. The inclusion of individuals’ genomic data in genomic datasets is critical to genomic research, and these datasets are most effective if shared widely. Genomic research consortia are an integral part of the genomic data sharing ecosystem, critical in facilitating data sharing among research groups. This article considers the property status of genomic data at various stages of the research life cycle, and the potential ‘ownership’ claims that may be made by various actors in data sharing networks. It does so by comparing the legal position with the findings of a study that examined policy documents and guidelines produced by international research consortia. This analysis enabled us to assess whether consideration of property interests is at the forefront of data sharing efforts, and if so, where such property interests are likely to reside.

## I. INTRODUCTION

There are likely to be few people who don’t feel some degree of connectedness to their genomic data. Genomic data is highly personal; it contains previously unascertainable information about a person’s health status, and is capable of unlocking knowledge about that individual and their close relatives. Questions continue to be asked about the extent to which genomic data can ever be truly deidentified, particularly if a person’s entire genome is shared.^1^ Viewed this way, it is unsurprising that individuals perceive that they should have some form of proprietary control over the use of their genomic data, that they ‘own’ their data. The same rationale can, and indeed has been made for health data more generally.^2^ However, the special and familial nature of genomic data, and its particular value as a research resource, set it apart from health data more generally.

In reality, an individual’s genomic data is unique to them only in a very small way. Much of the human genome is common to all individuals. Variants in genomic information across persons constitute a very minor part of the human genome—estimated at some 0.1 per cent—at least in composition if not in function.^3^ These variations between genomes account for approximately 300,000 variations per human. Even though not all are deleterious, it is these variations that are the point of interest in genomics, and the points of reference for database-wide studies and clinical diagnosis. Broadening the reference genome is a primary aim of genomic research, and maximizing data sharing will assist in capitalizing on this aim. The Human Pangenome Project is an exemplar of this drive to expand the human reference genome.^4^

Clinicians who have collected and analyzed genomic data obtained from their patients are likely to feel that they also have some right to own and control their clinical notes and diagnoses. Similarly, researchers will likely expect to have some rights relating to their research data and results, whether in the form of a simple right of attribution or greater control over how their data and results are used. Other researchers and entities engaged in follow-on research and technology development based on the results of genomic research will also expect some assurance that they will be able to recover their costs and control how their technologies are used. This is usually achieved by securing some form of proprietary rights over the technologies being developed, particularly in the form of patents or other intellectual property (IP).

The issues surrounding genomic data ownership and control are thus complex and not easily resolved. They become more intractable as data moves deeper into the research ecosystem. Of particular interest to us is the proprietary status of the various iterations of genomic data residing in data collections, generated in either clinical or research settings, and in many cases, having changed hands and format during their lifetime. As will be seen later in this article, the proprietary status of genomic data in this environment, increasingly referred to as the medical information commons (MIC),^5^ has begun to garner scholarly attention. The MIC has been defined variously as a ‘worldwide collection of genomic data that is generally available for public use’,^6^ and ‘a networked environment in which diverse health, medical, and genomic data on large populations become broadly available for research use and clinical applications.’^7^

As genomic data becomes more integrated into every facet of biomedical research, it becomes an increasingly vital component of the MIC.^8^ There are many ways that genomic data can be incorporated into the MIC, including through consortia established specifically for the purpose of facilitating collaborative genomic research. These genomic research consortia are becoming major contributors to the MIC and play an increasingly important role in facilitating large-scale data sharing.^9^ Our rationale for focusing on genomic research consortia is their prevalence in the research ecosystem, and their growth over recent years. They are, however, broadly representative of the range of other research data repositories that have been identified in the research ecosystem. Genomic research consortia have the responsibility to ensure that the genomic data they hold is shared and used ‘optimally’. But they also have the obligation to ensure that the data they hold is shared and used ‘appropriately’.

This article examines how the collection, storage, and use of genomic data within genomic research consortia are governed. The article first broadly identifies the salient characteristics of genomic data and then moves on to provide an overview of the common law position on the question of whether property rights or other proprietary interests could exist in that data. We focus attention on three geographical regions, the US, the UK, and Australia, both because they are particularly active in large-scale genomic analysis and also because they are rooted in the same common law system. Where relevant, we also mention European-wide approaches. The article then provides a brief summary of some of the other legal interests that might exist in the journey from data collection to use, and, if such interests do exist, in whom they might reside. The overview of property law and the summary of other relevant laws are provided to contextualizse the crux of the article, which presents findings on how genomic research consortia are currently dealing with these complexities through their governance arrangements, particularly focusing on the arrangements they use to address the interests and needs of donors, contributing researchers and users. The methodology employed is textual analysis of documentation pertaining to various genomic research consortia (including constitutional documents and data sharing agreements).

## II. GENOMIC DATA AND GENOMIC RESEARCH CONSORTIA WITHIN THE DATA ECOSYSTEM

Genomic data can take a number of different forms and vary in complexity. Broadly defined, genomic data refers to the DNA sequence information found in an individual’s cells. The term also encompasses genetic characteristics and information derived from tangible samples. A single genomic dataset may be as large as a person’s entire genome [ascertained by whole genome sequencing (WGS) or next generation sequencing (NGS)], or as small as a single gene, or anything in between. It is now possible to sequence an individual’s entire genome at relatively low cost; hence, genomic sequence data is proliferating and becoming an increasingly valuable research reference tool. Genomic sequencing might be undertaken for a number of reasons, whether for clinical or research purposes. A significant amount of genomic data is generated in the clinical context, both through specific sequencing of tumor cells (often referred to as somatic sequencing) and through more general sequencing of non-cancerous cells (germline sequencing).^10^ Research projects often utilize clinical data, although given the associated storage and transfer costs, together with concerns about data quality and reliability, samples are often re-sequenced for research purposes outside the clinical context.^11^ Hence, some genomic data is generated in the clinic and used for secondary research purposes, some is generated for specific research projects and subsequently used in other research, and some is generated for storage and use in future research projects.

In research terms, genomic data might comprise raw genomic data, annotated or curated data, or aggregated data. ‘Raw genomic data’, or bare sequence data, is contained in Sequence Alignment/Map (SAM) or Binary Alignment/Map (BAM) files. For this raw data to become useful in the research context, it needs to be matched against a reference genome to identify variants. A Variant Call File (VCF) is then produced, which lists deviations from ‘normal’ sequence data.^12^ The variants are then expertly classified by bioinformaticians. After removing known non-harmful variants, variants of significance are identified and interpreted, and an annotated version of the filtered VCF file produced.^13^

Annotated genomic data forms the basis of many genomic research consortia databases. It is at this point that the data is usually de-identified and collated. We refer to data contained in collections such as genomic research consortia as ‘aggregated data’, noting that it is often de-identified and aggregated prior to its inclusion. We note that Villanueva et al. use the term ‘aggregator’ to refer to a specific type of data aggregating entity in the research ecosystem. ^14^ Under their terminology, aggregators are defined as parties that ‘pool data from published studies, existing datasets, or from direct data submissions, and share an output, often through a browser.’^15^ In contrast, we use the term more broadly, including closed consortia, repositories, and data sharing initiatives relying on different forms of access within our definition of data aggregators, rather than treating them as separate categories. Aggregated data is the backbone of genomic research.

The purpose of many genomic research consortia is to facilitate population-wide research or personalized medicine by providing access to aggregated data to researchers and clinicians. Thus, further layers of data may derive through the use of data housed in consortia, or from the efforts of consortia-wide studies that engage multiple members of a consortium in particular research projects. Although genomic research consortia tend to adhere to some broadly consistent principles, particularly those developed by the Global Alliance for Genomics and Health (GA4GH), their governance structures are to a large extent individualized.^16^ GA4GH was established in 2013. It describes itself as ‘a policy-framing and technical standards-setting organization, seeking to enable responsible genomic data sharing within a human rights framework’.^17^

## III. NOTIONS OF OWNERSHIP AND PROPERTY IN RESPECT OF GENOMIC DATA

### III.A. Ownership, Property Rights, and Possessory Interests at Common Law

At common law, ‘ownership’ can be thought of as a legally recognizable property interest in a good, and the right to exercise power over that good.^18^ Fundamentally, ownership comprises a ‘bundle of rights’ over a thing.^19^ These rights, which provide the indicia for the existence of property rights, include, most relevantly, the right to possess, the right to exclude, the right to access, and the right to destroy.^20^ The concept of a bundle of rights has been recognized in case law in many common law jurisdictions.^21^ Grounded in Lockean theory,^22^ this notion of property treats goods as rivalrous products, which are capable of being possessed to the exclusion of others. However, possession may vest in more than one party at a given point in time, and it is not necessary that the full bundle of rights be present for some form of property right to subsist.^23^ Conveying property interests will permit an owner to deal with their property, including on a commercial basis.

In order to determine whether this bundle of rights exists in genomic data, it is first necessary to determine whether data is a ‘good’ capable of constituting property. ‘Personal property’ is defined as any form of property other than real property.^24^ It may include intangible goods but will not include knowledge or information although both may, in appropriate circumstances, be protected through IP rights.^25^ As to the types of interests the law might protect, personal property rights may subsist irrespective of whether or not the goods are capable of actual possession—but if not, the goods must be capable of being claimed or enforced by legal or equitable action. While ownership and actual possession of goods often coincide, a person in possession of goods may not own those goods but may nonetheless have other possessory interests. Although possession may accompany ownership, it may additionally constitute a lesser, substantive interest that may or may not be protected by law. For simplicity, throughout the rest of this article, we refer to the various property and possessory rights and interests as *proprietary interests*, recognizing that this term embraces a number of different rights and interests, not all of which amount to ownership.

Where full ownership of property does subsist, it may include legal possession or the legal right to possess. The legal right to possession enables an owner to exercise legal rights to reclaim a good despite not having actual possession of it at the time. The right to possess is also known as constructive possession—it may vest in the owner of goods where the owner has the right to possess, but not actual or legal possession.^26^ This might arise, for example, where an owner’s agent, bailee, or licensee has taken custody of the goods with the owner’s consent.^27^ In these circumstances, the owner retains some ongoing control of the goods, including the right to demand that the goods are returned to them. An owner will lose the right to possess upon transferring ownership or gifting the goods, resulting in loss of the right to demand that the goods be returned and to exercise other forms of ongoing control over the goods.

Difficulties in attributing ownership to individuals arise in identifying the point at which proprietary interests arise (if at all). Does genomic data, or derivative information,^28^ become capable of ownership once generated from a tangible sample? At what point does data cease to be about an individual and become part of an aggregated set of data, and are new ownership rights created at such time?

### III.B. Ownership of Human Tissue

Debates around ownership of human tissue have long preceded discussions around ownership of genomic data. The notion of property rights in tissue has been particularly perplexing, compounded when tissue is ‘donated’ either for organ donation or storage in biobanks for research purposes. Fundamentally, case law in the UK and Australia has determined that human tissue will be capable of being owned if there has been some application of work and skill (for example dissection or preservation).^29^ It is clear from this case law that preserved tissue, body parts, tissue excised during surgery, and cell lines derived from tissue might all amount to goods capable of attracting a possessory interest that may not amount to full ownership.^30^ In fact, separation from the body may be all that is required to establish possessory interest in tissue.^31^

The clear implication of this is that, before its removal from the body, human tissue is not owned by the individual in whom it resides. This inability to ‘own’ one’s own tissue is somewhat at odds with most tissue donation legislation, which relies on the law of gifts: the very nature of the concept of a gift (particularly as a vehicle for alienation of rights) implies recognition that an underlying property interest vests in the subject.^32^ This is supported by US case law that has determined that donation of tissue samples for research involves a gift from the donor to the institution, and that donors have no property interest in their tissue.^33^ Although such a gift is probably made with conditions attached, these conditions are likely limited to the right to withdraw samples rather than a right to control who uses the data or where it is stored.^34^ Conversely, the law of gifts has been argued to be an appropriate tool to govern the transfer of tissue samples for research, due to the fact that it would provide capacity to imply other conditions into the exchange.^35^

The operation of the law of gifts is contingent on recognition of some form of proprietary interest vesting in the ‘donor’. Although a possessory interest has been recognized in relation to sperm stored for reproductive purposes after cancer treatment^36^ or death,^37^ this does not necessarily pave the way for its broader application in human tissue pre-removal. There is a significant difference between the ‘sperm cases’, and cases involving donation of tissue for research. Most obviously, the donors or their beneficiaries in the sperm cases always intended to reclaim and use their stored sperm, as opposed to donors who have contributed tissue samples for research. In addition, it seems that a concurrent possessory interest existed in the laboratories.^38^ The potential scope of these decisions for tissue donated for research is not yet clear. What is clear, however, is that as soon as work and skill is exercised on tissue to generate a novel material (eg a cell line), the researcher will acquire some form of proprietary interest in that material (noting that other legal and ethical obligations must be complied with).

### III.C. Ownership of Genomic Data?

While proprietary interests in tangible materials may subsist in a researcher (or, more accurately, their institution), the derivation of genomic data from those samples creates a new set of issues. What follows is a brief overview of the arguments for and against vesting possessory interests over genomic data in particular parties. There is no clear consensus position in scholarly literature on the question of ownership, and little accord across judicial decisions. As a starting point, if proprietary interests arise upon the application of work and skill, as is the case for tangible tissue, this suggests that no individual can ‘own’ genomic data until it has, at a minimum, been sequenced and interpreted. However, there are other features of genomic data that further complicate the analysis. Genomic data is non-rivalrous and may in theory be stored and used indefinitely. On this basis the core attributes of data and tissue differ, creating difficulties in ascertaining whether proprietary interests exist, and whether genomic data is actually capable of being owned. Yet it is also highly personalized, containing information not only about the information source, but also about their relatives. Because it is so inherently personalized and such a powerful tool, calls for recognition of personal ownership of genomic information derived from individuals have intensified.^39^

Advocates of data ownership primarily argue that ownership should vest in the source of genomic data (or ‘donor’),^40^ on the basis that this would provide them with the capacity to assert control over their data.^41^ Ownership rights would, it is argued, give individuals more extensive privacy rights, more control over use, and the capacity to reap commercial benefits from use of their data. On a more emotive level, many agree that there is intuitive appeal in owning one’s genomic data.^42^

Those who oppose ownership of genomic data on the part of individuals generally do so on the basis that dispersed individual ownership would stymie research.^43^ The privatization of publicly funded research through its collation into valuable collections of data has led to public outcry and the closing off of significant genomic resources.^44^ If we take the concept of privatization one step further and consider vesting ownership rights in individuals, arguably this would result in an effective tragedy of the anticommons,^45^ because it would make creation of a MIC exceedingly difficult.^46^ Logistical difficulties in uploading data, in locating datasets, and accessing data in differing file formats are well documented^47^ and are likely to be exacerbated by individual ownership of genomic data, if it results in further fragmentation and assertion of rights. On this basis, some have suggested that property rights should vest in governments or public institutions.^48^

### III.D. Impediments to Recognizing Ownership Rights in Genomic Data

One theme that runs through the literature dealing with proprietary interests in genomic data is the notion that recognition of ownership rights or other possessory interests may add little value for those who would seek to assert rights over information.^49^ What individuals really seek in asserting ownership rights is either some kind of ‘benefit’, or some form of ‘control’ in dealings involving the asserted property.^50^ For donors, the value in being able to assert ownership of their genomic data lies in the fact that this might provide them with a legal remedy in the event that data is misappropriated or misused. As explained shortly, however, the conferral of ownership rights does not necessarily equate with increased control over data.

Another facet of the debate that complicates matters is the existence of IP rights, which may be claimed in order to facilitate commercialization. The very existence of the patent system creates unease among research participants, and the notion of commercialization could become a major impediment to research in its own right.^51^ IP rights could also be seen to be antithetical to the development of a research commons and the accumulation of research data in ‘common pool’ consortia. This notion is explored further below.

Added to these problems with the concept of ownership of genomic data, the aggregated/consolidated nature of research data means that disaggregation would often be difficult, and ownership claims would be collective rather than exclusive,^52^ and meagre at best,^53^ given the amount of sequence data required to make a useful, shared resource. The genomic data of one individual will often not be particularly useful; it is the aggregated resource that is valuable from a clinical and research perspective.

It has also been pointed out that imbuing genomic data with property status would not necessarily guarantee property-rule protection, which provides an owner with the capacity to prevent unconsented use.^54^ At best, it might only confer liability-rule protection, whereby property owners are not allowed to prevent unconsented uses, but may subsequently seek compensation.^55^ Indeed, recognizing the ‘property-like regime established under the guise of informed consent’,^56^ some have gone further and proposed a liability-rule style of protection that permits unconsented use but punishes researchers for ‘overstepping’ the bounds of permitted uses.^57^

Evans asserts that if proprietary interests in genomic data are awarded then, foreseeably, such data might be ‘taken’ (as a form of property) by the state under the doctrine of eminent domain,^58^ for use in public research. This reflects the notion that genomic data is a public good, generally donated for altruistic reasons, which should be widely available to researchers in order to maximize its utility. In Australia, compulsory acquisition of property is permissible under certain statutes, although the relevant statutory provisions generally apply in respect of infrastructure or public utilities.^59^ Whether this would include genomic data is debatable, although the argument advanced by Evans is that a possible ground for the compulsory taking of data might include a perceived need for complete datasets in order to develop research with a beneficial therapy in mind. The use of data for law enforcement or public health is already provided for in a number of contexts.^60^ Compulsorily acquiring genomic data would alleviate the problem of needing to negotiate with multiple ‘property owners’, and the threat of a tragedy of the anticommons.^61^ However the risk with such an approach is that it could remove any measure of control individuals have in their own data.

Another concern with recognition of proprietary interests in genomic data is that this could run counter to the desire of those who seek ongoing control of their data because transfers of property will result in transfer of ownership and/or possession (and therefore control). Data would cease to be owned or possessed by the individual from whom it is derived.^62^ Recognition of ownership rights in genomic data might work against the ultimate aim of ensuring control and privacy, on the grounds that once control is devolved by the donor, data may be dealt with in any way subsequent owners see fit.^63^

In short, there is significant ambiguity as to whether any party ‘owns’ genomic data and whether it is actually beneficial for them to do so. In particular, there seems to be little benefit from the perspective of the donor, if their goal is to protect their privacy and retain some control over how their data is used. Indeed, recognition of ownership of genomic data might have the perverse effect of making it easier for whatever control that may otherwise reside in donors to be given away.

## IV. OTHER INTERESTS OF PARTICIPANTS IN THE RESEARCH CONTINUUM

The previous section has illustrated the complexity, uncertainty, and divergence of opinion as to the application of property law in the context of genomic data sharing. Added to this, other property-like interests can attach to specific participants in the genomic data sharing ecosystem. Without attempting to be comprehensive, the brief analysis that follows provides a broad overview of some of the types of interests that genomic research consortia need to be cognizant of in establishing their governance arrangements. Specifically, it considers legal interests that might be attributed to donors of genomic data, clinicians, researchers who generate data and input this data into databases, consortia themselves, and end users of consortium data (researchers and their corresponding institutions).

It is noted that the analysis does not include the ‘groups between’—population groups such as ethnic groups, disease organizations/advocacy groups or families—who are also impacted by genomic research.^64^ Often, it is these groups that genomic research seeks to benefit and they should therefore be at the forefront of consideration in regulatory decision making.^65^ However, it is difficult to attribute property rights or other possessory interests to clearly definable but amorphous groups of this nature. It is for this reason that they do not play a part in the following analysis except to the extent that their data is included in the collective datasets held by genomic research consortia. Nor do we specifically consider the data sovereignty of First Nations peoples because it would be impossible to do justice to this issue within the parameters of the topic under discussion.

### IV.A. Sui Generis Donor Ownership Rights and Privacy Rights

There has been considerably more debate in the US than in other jurisdictions as to whether proprietary interests in genomic data might vest in particular individuals and/or entities. Several US states have enshrined limited genetic ownership rights in statute.^66^ Under the laws of these states, genetic information is the property of the person from whom it was derived. There is also some case law emanating from two of these states where courts have been willing to recognize proprietary interests in donors of genetic information. In *Peerenboom v Perlmutter*,^67^ genetic material obtained surreptitiously for use in a lawsuit was held by a Florida state court to have been converted based on the property interest of the parties from whom it was taken. Similarly, in *Cole v Gene by Gene, Ltd*^68^ an action alleging a breach of privacy legislation against a direct-to-consumer (DTC) genetic testing company was brought after the company shared the data with third parties. A summary motion to dismiss by the company on the grounds that Cole had not suffered a compensable injury was rejected by an Alaskan district court on the basis that individuals have a lawful property interest in their genetic data.^69^ The scope of legislation in other states recognizing property interests in genomic data remains untested in the courts at the time of writing. The fundamental position, then, is that there is inconsistency across US states as to whether donor ownership of genomic data exists.

In the other jurisdictions under consideration (the UK and Australia), this notion of donor ownership of genomic data has failed to gain traction, either through common law principles^70^ or in the form of sui generis legislation. Although the idea of ownership of digital data more generally has been mooted in Europe, there has been no concrete move to achieve this.^71^ Were such a right to be created, it is unclear whether this would extend to genomic data. Donors have, however, been accorded extensive rights under the recently enacted General Directive on Data Protection (GDPR),^72^ which has been implemented in the UK through the *Data Protection Act 2018* (UK). The GDPR also imposes stringent controls over the collection, processing, and storage of data, and European citizens have a fairly unfettered right to access their personal data (including health data). Data portability mechanisms provide donors with the capacity to ‘transact’ with, and make decisions to, relocate their data.^73^ Although privacy rights such as those vested by the GDPR provide some form of possessory interest, they do not align neatly with the notion of property interests in that privacy law seeks to protect many more conceptually diverse interests than the law of property.^74^

As in other jurisdictions, Australian patients have a statutory right to access their medical data (including raw genomic data),^75^ but no proprietary interests over records created by a healthcare practitioner.^76^ State and territory privacy laws, freedom of information legislation, and the Australian My Health Record^77^ have overridden the common law position that patients have no inherent right under a clinician’s fiduciary duty to access their medical records.^78^ There is no authority dealing with the ownership of genomic data, or even whether it is considered property. However, rights somewhat equivalent to those attaching to property are provided through the privacy and consent regimes.

### IV.B. Clinician Notes

Under the law of each of the jurisdictions being examined, clinicians own the material form of medical records they create. In the US, property rights in the clinical records subsist in health providers through the Health Insurance Portability and Accountability Act (HIPAA) Privacy Rule. They, in turn, have a statutory obligation to disclose information contained in those records to patients (and provide copies thereof) upon request.^79^ This means that medical imaging scans, for example, remain the property of the facility that generates the images.^80^ In the UK, although copyright over clinical notes lies with clinicians, patients have extensive rights to access medical records under the GDPR via the *Data Protection Act 2018*. It has been pointed out these rights appear to be remarkably similar to proprietary interests.^81^ Similarly, in Australia, copyright over medical records created by a clinician resides in the relevant practitioner,^82^ but patients have a clear right to access that data.

Although it seems clear that clinicians ‘own’ clinical notes into which they have intellectual input, this will not extend to raw genomic data generated through NGS or WGS, nor would it extend to annotations and interpretive material in respect of raw genomic data, unless added by the clinician as a result of intellectual effort. Liddell et al. conclude that this would appear to be the case at least in the UK, as well as some other European countries.^83^

### IV.C. Genomic Sequences

There is no legal precedent in any of the jurisdictions considered that has tested whether *ownership* vests in researchers, or the generators of genomic sequence data. Attributing ownership to researchers would make a degree of sense where intellectual effort has been expended generating the data and interpreting it to ensure utility,^84^ such data corresponding, in this sense, to the evolution of body tissue into a thing that is capable of ownership through the application of work and skill. Although this could perhaps be said to be the case for interpretative material/annotations on data, ^85^ the automated nature of genome sequencing means that generated data in its most fundamental, raw sense will not meet the threshold for ownership.

The question of whether patents can be granted for gene sequences remained unanswered for many years. Following superior court decisions, it is now clear in the US and Australia that patents are not available for gene sequences in their natural form.^86^ Under US law, a gene sequence has been held to be a product of nature and not patentable where it has simply been isolated.^87^ On the other hand, human-made sequences containing the same informational content (notably cDNA) may be patentable provided that they satisfy the other patent criteria on the basis that they are markedly different to the naturally occurring sequences.^88^ Under Australian law, patent protection is not available for either naturally occurring or human-made gene sequences because both comprise mere information that has simply been discerned by the relevant researcher.^89^ The more permissive European law permits the patenting of gene sequences provided industrial applicability can be demonstrated.^90^

What does all this mean for the products of WGS and NGS? In line with the principles derived in the US and Australia, WGS or exome sequence data of itself is likely to be viewed as non-patentable, unless some human intervention or ingenuity in its generation or interpretation can be demonstrated. This intervention is likely to manifest in the form of identification of variants from the wild-type genome—it is in respect of the identification and annotation of novel segments of the genome indicating a predisposition to disease that patent protection is likely to be sought. Although there have been a number of lower court decisions in the US seeking to set limits around the expansive nature of the higher court decisions, there remains considerable uncertainty^91^ and concerns about the adverse consequences for molecular diagnostics.^92^ Even so, the results of a recent empirical analysis ‘suggest that molecular diagnostic patents are harder to get but they are still being applied for and granted, with their narrowed scope making them less likely to block follow on innovation.’^93^

Copyright protection may be available as an alternative. Copyright will not protect facts, but is available to protect data in some instances. Copyright protects original literary or artistic works but, in each of the jurisdictions under consideration, is unlikely to protect genome sequence data without more. Precisely what copyright may protect in this context is far from clear.^94^ Copyright requires clear identification of the author of a protected work, and evidence of originality. Under Australian law, for instance, copyright may subsist in medical records in some instances, provided the requisite evidence of expression and authorship can be established: this intellectual effort may be minimal,^95^ but this is unlikely to extend to gene sequences because there is no opportunity for expression in simply restating the letter sequence that constitutes a gene.^96^

A similar position is evident in the UK^97^ and the US,^98^ although some have argued for the availability of copyright protection for engineered genomic sequences,^99^ and other emerging technologies.^100^ While the fundamental position that gene sequences are not copyrightable is sound,^101^ in the genomic data context, there are many technological situations where human intervention sufficient to amount to authorship is evident: the synthesis of DNA and alteration of genes through gene therapy and genome editing techniques are good examples. Even in respect of WGS, it is conceivable that copyright may subsist in interpretive data and annotations made by researchers and bioinformaticians, recognizing that a threshold level of data analysis (as opposed to machine-generated data) is undoubtedly required in order for copyright interests to arise. Such works are akin to the recording of medical information in that they evidence some degree of expression and originality on the part of the researcher undertaking the interpretation.

There is a real question around the scope of protection copyright would provide if available. Copyright over, for example, an engineered genome sequence or WGS would protect against direct reproduction by others. Given that sequence data is run through highly specific interpretive pathways unique to the ethics protocols of individual research projects, copyright would provide limited protection for the interpretive data produced in an individual project, but would not preclude reuse of genomic data to provide alternative interpretations.

### IV.D. Compilations

The compilations of data present in genomic research consortia present further issues for consideration. Depending on the nature of data collections held in these consortia, copyright may attach to them in the form of compilations. In the US, copyright may attach to compilations of data where the consolidation of the data involves creative effort.^102^ Under Australian law, there have been judicial decisions that suggest copyright will not subsist in computer-generated compilations where there is minimal creative human input,^103^ although as we have argued above, the interpretation and annotation of genomic files may constitute sufficient intellectual input to qualify for copyright protection. The collation of datasets adds an additional layer of expression. In other jurisdictions (for example Canada), compilations of data do seem to attract copyright protection.^104^ There is thus marked jurisdictional inconsistency with regard to copyright protection in compilations of personal data. Similarly, there are few jurisdictions that accord sui generis protection to compilations contained in information databases. While such a regime exists under European law,^105^ it is not replicated in other jurisdictions such as the US or Australia.

### IV.E. Outputs Produced Using Consortium Data

As for the outputs of research utilizing consortium data, these may be the result of consortium-wide efforts, but they will often be the work of individual researchers or research groups (see below). Consortia facilitate large-scale research programs; consortia members might utilize consortium datasets for use in a variety of research projects. Research outputs are hugely variable, and will range from knowledge exchange to publications, patents, building capacity, and development of evidence-based solutions.^106^ Given the focus of this paper, we confine our consideration of ‘outputs’ to publications and IP rights, recognizing the many important functions of research collectives in performing other functions.^107^ An early study of eight European consortia discovered varying methods of distributing IP rights in research outputs emanating from consortium data. These ranged from individual ownership to collective ownership, depending on the nature of the relationship between consortium members.^108^ It has also been argued that, in the absence of specific contractual terms, proprietary interests in data derived from consortium-wide efforts are likely to reside in the consortium as a whole.^109^ The public release of data may, however, impact on novelty and thus capacity to patent, and would need to be managed accordingly.^110^

Outputs produced by individual researchers (rather than the consortium as a whole) using aggregated data sourced from a consortium may be governed by terms in consortium agreements or data sharing agreements (discussed further below). It has been argued that where there are no such clauses, outputs should be owned by the consortium as a whole.^111^ However, should copyright subsist in these outputs, it will reside in the author unless the relevant governing documentation provides otherwise. And if patents are sought for outputs satisfying the patentability requirements, these may likewise be owned by the generators of those outputs, absent agreement to the contrary.

Debates about ownership are further complicated by the relationships between individuals generating the outputs and their employers and funders. In the US, there is legislation that specifies that as a general rule, federal funders will not claim ownership of the outputs of research they fund, but leave decisions about pursuit and ownership of patents to the universities and federal laboratories they fund (while retaining march in rights).^112^ Although other jurisdictions do not have equivalent legislation, they tend to follow the same policy. The situation is likely to be different where individuals generating outputs are funded by arrangements with the private sector. In such circumstances, they may be required to assign ownership to the funder.

Although US legislation clarifies the situation regarding ownership at an institutional level, the 2011 Supreme Court decision in *Stanford v Roche* shows that this does not change the common law position that university employees are the original owners of patent rights arising from their work.^113^ As such, unless there is clear assignment of those rights, they continue to vest in the individual output generator. Likewise, the Australian case of *University of Western Australia v Gray* emphasized that universities need to have clear contractual provisions or policies vesting them with rights to employee inventions.^114^ The more recent UK decision in *Oxford University Innovation Limited v Oxford Nanoimaging Limited* shows that the situation becomes even more complex when the output generator is an intern or PhD student.^115^

All of this is not to say that property cannot be jointly owned. In the case of an intangible such as data, the ‘asset’ is inexhaustible in the sense that it can be used simultaneously by multiple parties and reproduced indefinitely.

## V. GOVERNANCE OF THE DIVERSE INTERESTS IN GENOMIC RESEARCH CONSORTIA

What we have seen in the above analysis is a highly complex legal landscape, including: uncertainties in legal protections available to the various stakeholders; potential competing proprietary interests; and lack of harmonization in legal protections globally. To summarize, there is little doubt that donors are unlikely to have proprietary interests over their genomic data once it has been contributed to a research study. Even if it were the case that proprietary interests did reside in the donor, a decision on the part of that individual to consent to the donation of their data to a research study may indicate a transfer of property and/or possessory interests. The difficulty in separating individual-level data from aggregated data is likely to mean that donors are unlikely at that point to be able to request the return of data or otherwise exercise control over that data.

Proprietary interests in research using genomic data may arise in respect of researchers. They may take the form of property interests in the traditional sense, or IP rights. The most likely candidates in this respect are copyright over the interpretive results and annotations of sequence data and patents over the outputs of research using the data. Consortia are unlikely to acquire property interests over contributed data or outputs generated by individual researchers or research groups, but may be better placed to assert proprietary interests over consortium-wide efforts. Ownership of the outputs produced using consortium data depend on the circumstances under which the outputs are produced. The greater the effort expended by individual users, the less likely that proprietary rights could be claimed by other members of the consortium. Ownership of these outputs is also dependent on the contractual relationships between the individuals who generate them and their employers and funders.

Consortia are responsible for managing collection, storage, and use of genomic data in ways that address these complexities. This all points to the need for consortia to have well-articulated governance arrangements. In order to ascertain the extent to which genomic research consortia have risen to the challenges described above, we undertook an examination of consortia governance documents. We sought to ascertain the ways in which consortia recognize the boundaries of ownership set out in the previous sections, and respond to some of the legal uncertainties and differences between jurisdictions that we have highlighted.

It has been observed by Contreras that uncertainty as to where ownership of data resides has in some cases resulted in contract being a primary method for establishing the legal foundations of research relationships, including acceptable uses and data release.^116^ More broadly, while the existence of proprietary interests may increasingly be determined by the laws and doctrines explored above, can contractual arrangements be effective to transfer these interests once they are in existence? The following section reports on an analysis of the terms and conditions in consortia documents to ascertain what provisions as to ownership of genomic data have been included.

### V.A. Methodology

A list of genomic research consortia was compiled by conducting searches for relevant bodies in literature emanating from consortia. The authors’ existing library of over 800 academic articles and other documents relating to genomic data sharing was used to conduct preliminary searches for information on genomic data sharing, with a further search undertaken on PubMed using the term (consortia[Title]) OR (consortium[Title]). This search yielded 7000 results. A search to refine the results was undertaken by narrowing the search terms to: health/genetic/genomic consortia. Eighty-nine documents were selected and compiled for analysis in 2019 as a result of these searches, on the basis of their particular focus on data sharing arrangements. These documents were supplemented by more recent literature published during 2020 to 2022. We do not assert that this catalogue of results was an exhaustive collection of articles relating to genomic data sharing, but rather a sufficiently large sample of the literature (and genomics research consortia) to identify research consortia and therein, commonalities and divergences in practice between consortia.

Separately, a library of foundational articles and governance policies, guidelines, agreements, and other documentation made available by individual genomic research consortia was compiled to enable detailed analysis of their specific governance arrangements. Originally drawn from a catalogue of genomic data sharing initiatives compiled by GA4GH (no longer available on the GA4GH website), this library was then supplemented by articles from our PubMed search that focused on specific consortia, and results from other publicly available lists of consortia, including: data sharing repositories available on the National Institutes of Health (NIH) website;^117^ repositories recommended by Nature; and a list of driver projects on the GA4GH site.^118^ Rather than being an exhaustive list, this list was intended to comprise a comprehensive dataset of entities that self-identified as genomic research consortia. We excluded entities from our initial list on the following grounds.

- Entities that do not engage in research into genomics at the molecular level (eg the Human Cell Atlas) on the grounds that their primary focus is not genomics;
- Genomic repositories, archives, and databases that store genomic data, such as the US-based database of Genotypes and Phenotypes (dbGaP),^119^ as they were not established for the specific purpose of undertaking genomic research, but rather to facilitate sharing of research data. However, genomic research consortia that deposit data in these repositories may be bound by their data access policies, and in this respect their policies are relevant to our analysis;
- Entities whose only role is to analyze data and are not engaged in collaborative research; and
- Genomic standard setting and funding bodies (eg NIH and the UK Wellcome Foundation), and bodies with active involvement in genomics research.^120^ The model data sharing policies and other documents developed by these bodies remain relevant to our analysis in that they they are often adopted by genomic research consortia.

We identified 102 consortia in total. This dataset was subsequently analyzed separately and reduced to 97. We removed four consortia that were revealed to not actively involved in genomic research, and one consortium that had been dissolved some years prior to our project and had very little governance information available. Other consortia in our dataset have been finalized, but governance information remains available. One entry was divided into two (International Cancer Genome Consortium (ICGC); International Cancer Genome Consortium - Accelerating Research in Genomic Oncology (ICGC-ARGO)) because the nature of the consortium altered with the establishment of ICGC-ARGO. The final number of consortia in the dataset was 98.

Our research and the research of other scholars has shown that genomic research consortia may take a number of different forms in relation to data sharing.^121^ The type of access arrangement adopted by a particular consortium is a very relevant factor when considering the possibility of claiming proprietary interests; capacity to claim property interests over data made available on an open basis is likely to be very different to the capacity of those involved in a small, members-only consortium. We have defined ‘consortium access (CA) arrangements’ as arrangements that require members to share contributed data with other consortium members on a reciprocal basis.^122^ This type of arrangement does not compel members to share outside the consortium.^123^ Members are often collaborators, and this form of consortium is usually set up as a vehicle for collaboration between particular parties with a specific research focus. By contrast, open access (OA) consortia make data publicly available to any potential user without restrictions on use.^124^ Consortium members are not afforded privileged access arrangements.

Some consortia make data available to outside users, but on the basis that they register prior to being afforded access. Registered access arrangements require potential data users to acknowledge adherence to terms of use attached to data through a simple online agreement.^125^

Managed access (MA) initiatives are often larger-scale arrangements,^126^ which do not give preferential access to consortium members. Rather, they provide access to specified sets of data by consortium members *and* external users upon application and/or through a data use agreement.^127^ Access requests are often managed through a Data Access Committee (DAC).

Finally, some consortia are hybrid in nature, allowing OA to certain datasets but requiring formal application for access to others. Tiered access (TA) consortia are classified as such because they allow consortia to use tailored levels of access based primarily on the type of data to which access is requested. Aggregated data might be openly available, while other levels of data may be sensitive enough to warrant MA or even CA.^128^ Within the TA category, up to four access tiers may exist for a single consortium, depending on the type of data in question.^129^

Forty-two consortia in our dataset are NIH-funded or affiliated, and a further 16 are affiliated with other public or private bodies such as the Wellcome Trust, Genomics England, the NIH, the Broad Institute, and the Bill and Melinda Gates Foundation (or a combination of these organizations). The vast majority of these consortia (52/58) adopt TA structures, with the remainder adopting either MA or OA models.

Once the consortia had been categorized, content analysis of all publicly available documentation produced by each consortium was undertaken.^130^ This included consortia websites, foundational documents, policies, publicly available contracts and agreements, and published articles documenting the foundation, aims, and structure of particular consortia. From these documents, we were able to undertake detailed analysis of the operation of relevant consortia. Among the terms examined were terms pertaining to ownership of consortia data, provenance of data housed in consortia and shared between consortia members, authorship and attribution, and IP. Given that 58 of the total number of consortia we examined are NIH, Wellcome, or other public/private body affiliated, the data sharing policies of the NIH^131^ and Wellcome^132^ are also particularly significant and were considered as part of this analysis.

A limitation of this analysis is that we relied very much on publicly available documentation, which was sparse in many cases. It is likely the data we have gathered does not tell the full picture, and that practices within consortia frequently take the place of written documentation. Although it is likely that a good number of consortia adopt the policies produced by standard setting bodies such as GA4GH and the NIH, it is often difficult to locate evidence of adherence to such policies in publicly available documentation. Further, assessing whether consortia are OA, registered, MA, TA, or whether access is restricted to consortium members is not always straightforward. In many cases this was clearly stated, but in some cases, judgement was required on the basis of evidence gathered and there were several instances where we made subjective assessments which formed the basis for the conclusions reached.^133^ A Supplementary File containing a summary of our findings has been included.

### V.B. Provisions as to ‘Ownership’

As the background discussion in Part IV demonstrates, there is considerable uncertainty surrounding ownership of genomic data, and this legal uncertainty is amplified once data is aggregated and/or shared. Navigating ambiguous territory such as this invariably presents challenges for consortia in establishing and enforcing suitable policies around data contribution, storage, and use.

To our knowledge, there has been no empirical study that has sought to consider whether genomic research consortia make provision as to ownership of the genomic data that they house. There has been consideration of IP terms in the policy documents of large-scale biobanks,^134^ which is of some relevance because a number of large biobank policy documents were reported in that study to contain very inclusive notions of IP that incorporated all forms of IP, knowledge, and in some cases data and databases.^135^ We consider terms relating to IP ownership of genomic data below.^136^ In this sub-section, we consider the particular issue of ‘ownership’ terms more generally, noting that this more expansive term might be intended to incorporate proprietary interests generally rather than to deal specifically with IP interests.

In this study, we sought to determine whether consortia documentation makes explicit reference to ownership of, or other proprietary interests in, this genomic data. Our intention here was to consider terms that purported to deal with ownership interests on a broader level than IP interests. One earlier study considered whether DTC genetic testing companies claim ownership of genetic information in their service terms.^137^ The study, involving 90 DTC testing companies, found that companies tended to claim commercialization rights rather than explicitly claiming ownership rights over consumers’ DNA. The content of such terms and conditions is important as they may place limits on the rights of consumers to recover in the event of misuse of their data.^138^

Unsurprisingly, in our analysis very few consortia documents (five in total) contain any provision for recognizing ownership of genomic data. Only one document associated with a consortium makes explicit reference to ownership of genomic data housed in that consortium. The International Cerebral Palsy Genetics Consortium contains detailed provision for recognition of ownership in its draft Data Transfer Agreement.^139^ The Primary Investigator’s Institution is named as a party to the agreement by virtue of being the ‘Data Owner’, and a clause of the agreement entitled ‘Ownership and Use of Data’ acknowledges that data being transferred from the consortium is the ‘property’ of the Data Owner, and that data is transferred to the consortium via a non-exclusive, perpetual, transferable, fee-free license.

Aside from this example, recognition of ownership in genomic data in this overt form is rare. We identified several agreements containing some allusion to ownership rights although it was not always clear what this term meant. For example, the Exome Aggregation Consortium (ExAC) [now the Genome Aggregation Database (gnomAD)] contains acknowledgment that contents of a database, or database itself, can be covered by other rights, including contracts and data protection rights. The Memorandum of Understanding (MOU) states that ‘Participants’ (contributors of sequence data) are ‘…not limited in any way in the use and publication of their own data.’^140^ The Solve-RD Code of Conduct also hints at this. In encouraging joint authorship by users of data, it stipulates that: ‘If the primary data is the key to discovery, a key authorship position should be discussed with the owner of the primary data.’^141^

There are many terms used to refer to the contributor of data, such as ‘Data Controller’, ‘Data Submitter’, or ‘Data Provider’. In some cases, there is an inference that this party has a proprietary interest in submitted data (through, for example, references in data sharing policies to ‘their’ datasets).^142^

It is clear from this analysis that few consortia see value in overtly acknowledging proprietary interests in consortium data given the low probability such interests will be legally recognized. Following from this, our expectation was that we were more likely to find indirect recognition of the interests of various stakeholders in the data sharing ecosystem.

### V.C. Representations as to ‘Provenance’

A larger number of consortia documents (ten in total) reflect some intention to ensure that accurate chains of custody in respect of consortium data are maintained. Although many consortia have processes in place to ensure data is validated, it appears that the purpose of validation is to ensure data is submitted in a consistent format rather than confirming the source of the data. There is an implicit assumption that the acceptance and use of data from approved institutions with study approval processes in place ensures strong data provenance from these data generators. The most common assurance of data provenance is generally through consortia requirements to ensure that appropriate ethics approval and consent approval processes have been obtained and adhered to by data contributors.^143^

As to maintaining the chain of provenance during the process of data distribution, a number of consortia refer to themselves as providing ‘managed’, ‘controlled’, or ‘closed’ access. Verification of legitimate users under these models is an important aspect of ensuring data quality and source. Limiting access to data files to approved users^144^ and requiring attribution in publications^145^ assists in tracking data.^146^ The gnomAD consortium, for example, in its Terms of Use stipulates that there is no legal requirement for those using data released by gnomAD to give attribution. However, attribution is encouraged on the basis that it ‘…supports future efforts to release other data. It also reduces the amount of “orphaned data”, helping retain links to authoritative sources.’^147^

This statement is a concerted reference to ensuring proper chains of custody are developed. Several other consortia made specific reference to processes for maintenance of accurate records of provenance. Genome England, for example, is explicit in its Protocol for the sharing of data pursuant to the 100,000 Genomes Project:^148^

8.5.6 Data from sample acquisition site

Local sample metadata will be required to be submitted in advance and alongside the DNA samples, to enable sequencing and annotation. Data will be submitted in accordance with the NHS GMS requirements or equivalent.

8.5.7 Data from national NHS and other sources

Genomics England expects to collect other health data on its consented patients into its data centre, to link outcomes data to the WGS and clinical data already held. This may involve ongoing data delivery from other organisations, including but not restricted to: NHS Digital, the CPRD or other sources of primary care data, Public Health England, disease registries, screening programmes and patient communities.

All organisations operating within the pipeline need to provide monitoring and reporting functionality to facilitate the tracking of samples and data end-to-end.

Likewise, the International Human Epigenome Consortium (IHEC) provides in its governing documents that the release of metadata and prepublication data to assist users in their analyses is encouraged. However, data users should ‘[a]ccurately and completely cit[e] the source of prepublication data, including the version of the data set (if appropriate)’; and ‘[b]eing aware that the released prepublication data may be associated with quality issues that will be later rectified by the data producers’.^149^

The ENCODE consortium provides in its Data Use, Software, and Analysis Release policies that:

All analysis results and data analysis products generated by the ENCODE consortium that will be of broad use to the community must be registered at the DCC under unique accession numbers as soon as they are stable, and certainly no later than the time of manuscript acceptance.

The PsychENCODE consortium likewise contains detailed provision for ensuring the traceability of data:^150^

Recipient will provide Genetic Data, indexed by NIMH subject ID number and cell-ID number, in the electronic format specified by dbGaP. When genotyping has been conducted, DNA marker names and allele sizes in base pairs will be provided for each individual subject, as indexed by NIMH subject ID number. Descriptive information about each typed marker, including marker name, allele sizes in base pairs and corresponding frequencies, relative distances in Megabases and in Centimorgans, marker heterozygosity, and the source of information used to determine map location, will also be provided. Recipient also agrees to submit to the Center all data relevant to the establishment of family structure as determined from laboratory analysis, at the time such determinations are made.

ICGC-ARGO similarly provides the following guideline in its Data Release Policy in relation to clinical data: ‘Member projects and leads should facilitate a process for the demonstration of traceability of data, including Good Documentation Practices…’^151^.

Aside from these limited examples, we found no further mention of provenance. Other consortia do require an undertaking from data users not to distribute data to users who are not authorized by the consortium to access data,^152^ which indirectly assists with maintenance of provenance as data becomes more heavily utilized. In addition, NIH^153^ and Wellcome^154^ policies contain broad statements that assist in ensuring data quality is front of mind for data contributors, and data quality was referred to either overtly or implicitly in the policy documents of a number of other consortia.

Ensuring the traceability and quality of data is undoubtedly a critical aspect of maintaining a degree of control over data. Whether control manifests in data contributors, consortia, or data users is contingent on the manner in which relevant provisions are framed. Although we found a small number of terms specifically referencing data provenance, in most cases, relevant terms make it incumbent upon data users to ensure accurate record keeping and acknowledgment of data sources.

### V.D. Attribution

Attribution of data creators is another mechanism through which contributors of data may receive acknowledgment, and through which provenance of data may be tracked. Arguably, attribution is a strong incentive to share data, and the impact of a perceived loss of control by data contributors once data has been shared can be effectively mitigated in that the provision by consortia of assurances as to attribution is one way of encouraging data sharing by researchers.^155^ Publication and attribution are perhaps the most critical aspect of genomics research. Indeed, embargo and attribution provisions have become an increasingly important component of data sharing principles during the evolution of the MIC.^156^

We observed a greater prevalence of terms or statements addressing attribution than terms regarding ownership or provenance. In total, 19 consortia documents include a direct statement that the contributor of datasets, the consortium, or both, should be acknowledged in any resulting publication. References to attribution or acknowledgment of data contributors are primarily located in data submission and data use agreements. Attribution is mandated in some instances and encouraged in others. For example, NIH-funded consortia such as the Clinical Sequencing Evidence-Generating Research Consortia (CSER) adhere to NIH data sharing policies which provide that investigators using data *must*:

Acknowledg[e] in all oral or written presentations, disclosures, or publications of the contributing investigator(s) who conducted the original study, the funding organization(s) that supported the work, the specificdataset(s) and applicable accession number(s), and the NIH-designated data repositories through which the investigator accessed any data.

A publication policy specific to CSER further provides that:

The CSER SC may decide that publications may be written on behalf of CSER investigators collectively (eg a CSER marker paper). These manuscripts may arise from participation in the Consortium or involve analysis of data from all CSER sites.

Similarly, provisions specific to the NIH-funded GoT2D Consortium provide:

Approved Users are strongly encouraged to publish their results in peer-reviewed journals and to present research findings at scientific meetings. Approved Users agree to acknowledge the Submitting Investigator(s) who submitted data from the original study to an NIH-designated data repository, the primary funding organization that supported the Submitting Investigator(s), and the NIH-designated data depository, in all oral and written presentations, disclosures, and publications resulting from any analyses of controlled-access data obtained through the attached DAR. Approved Users further agree that the acknowledgement shall include the dbGaP accession number to the specific version of the dataset(s) analyzed. A sample acknowledgement statement is provided for each dataset in the Addendum to this Agreement.

In like vein, the Wellcome Sanger Institute (WSI) (a number of consortia including the Transforming Genetic Medicine Initiative are funded by WSI) provides that researchers should be appropriately credited for their contribution to data collection.^157^

By contrast, the ExAC consortium managed by gnomAD provides as follows:

Citation in publications. We request that any use of data obtained from the gnomAD browser cite the gnomAD flagship paper and any online resources that include the data set provide a link to the browser. There is no need to include us as authors on your manuscript, unless we contributed specific advice or analysis for your work.

Some consortia are more circumspect in requirements as to acknowledgment and some directly require that consortia and data contributors *not* be acknowledged. The ENIGMA Corporate Policy, for example, provides that data sharing agreements ‘will specify that the ENIGMA name is not to appear in any clinical reports, although the corporate entity is free to mention their ENIGMA membership and data contribution on their website.’

It is difficult to reach firm conclusions as to whether the requirements of consortia as to attribution or acknowledgment are contingent on the nature of a particular consortium and whether data access is open or managed. Of the 19 consortia that either require or request attribution, there is very little variation in data access structures; a majority of consortia that either contained a term requiring attribution or recommended attribution have tiered structures. This aligns with TA comprising the greatest number of consortia examined, as demonstrated in Table 1, and with consortia appearing to be most likely to require attribution as a condition of access to data, perhaps because they exhibit greater control over submitted data.

**Table 1:** Categorization of collated consortia

CA	OA	Registered access	MA	TA
8	5	1	8	76

Of those consortia that are characterizable as TA, a majority fit into the broad category of being a combination of CA during an embargo period, MA for particular datasets, and OA for summary or aggregated data, in line with the requirements laid down by the NIH data sharing policy (13 of these 19 consortia were affiliated with the NIH, Wellcome, or other public bodies). Hence, we can conclude that the absence of specific provision in consortium documents does not paint the whole picture, and attribution of data contributors by those using consortium data is required. The NIH Data Sharing Policy (2014)^158^ contains a specific requirement to this effect, as does the Wellcome Policy.^159^

There is certainly scope for increased recognition by governing bodies of remaining consortia, of the importance of attribution, and the incentivization function it potentially performs (Table 2).

**Table 2:** Access arrangements for consortia requiring data attribution

CA	OA	Registered access	MA	TA
0	1	0	3	15

### V.E. Intellectual Property

We also examined whether particular consortia make any representations about IP ownership among consortia members. Our expectation was that terms making provision for members to claim IP would be prevalent in consortia, providing a more effective and less contentious mechanism to tackle the uncertainty surrounding ownership of genomic data than using language of ‘property’. In this context, the findings in the study by Jordan, Liddicoat, and Liddell are of interest, in that their study reports extensive use of terms relevant to claiming IP rights (almost 90 per cent of biobanks), in documents governing the operation of large human biobanks.^160^ Most of these terms purported to lay down a position in relation to background IP.

Twenty-six consortia make explicit reference in consortia documents to IP, while two further consortia made more general references to ‘commercialization’. It should be emphasized that we are not representing that the remaining consortia did not permit, encourage, or discourage commercialization—there is just no overt reference to IP protection in the governing documents of these consortia.

Where present in policy terms, ‘intellectual property’ is in most instances defined broadly. Most provisions encompassed IP in general rather than particular forms of IP (patents; copyright; confidential information), with a relatively small number referring to a distinct IP right. Seven consortia made specific reference to copyright, but all bar one^161^ couched this in terms of recognizing data contributors’ ‘copyright and other IP’ in contributed data.^162^ Five consortia referred specifically to patents in IP terms in consortia documents, all referencing patents arising from use of consortium data.^163^

This is in contrast with the findings of Jordan, Liddicoat, and Liddell, which also reported on the expansiveness of the definitions employed to describe IP. A notable finding from that study is that of the 11 biobanks that defined IP, most employed very broad definitions to include virtually every variety of protectable subject matter in existence. The consortia in this study defined IP relatively narrowly in comparison, although admittedly a number used catch-all phrases that are arguably all-inclusive to capture all forms of subject matter. It is salient, however, that research consortia housing data appear to define IP far more conservatively than biobanks.

There are various ways in which consortia documents containing representations as to IP protection dealt with it; these can be broadly categorized into provisions that dealt with whether IP may be claimed over outputs, and provision as to how IP over contributed data will be treated. In relation to the first issue, the NIH provides in its Genomic Data Sharing Policy that:

NIH encourages patenting of technology suitable for subsequent private investment that may lead to the development of products that address public needs without impeding research…NIH encourages broad use of NIH-funded genomic data that is consistent with a responsible approach to management of intellectual property derived from downstream discoveries…NIH discourages the use of patents to prevent the use of or to block access to genomic or genotype–phenotype data developed with NIH support.

The NIH-funded consortia analyzed in this study are required to comply with this policy position. As such, a number of these consortia make no further mention of IP in consortia policy documents. However, this is not universal, and both-NIH-funded and other consortia produce documents that contain provisions in relation to seeking IP protection. While some align with this NIH guidance, others deviate from it. A number of consortia (six in total) specifically provide that consortia members/users of data may claim IP over research outputs produced using consortia data. A smaller number (four) provide that any IP produced would be the property of either the consortium or the funding bodies. In the case of one of these, provision is made for joint ownership of IP over outputs by both the funding body and individual users.

At the other end of the spectrum, five consortia documents specifically provide that data users must not claim IP rights over outputs produced using primary data. Documents in respect of five other consortia provide that although it may be necessary for users of consortia data to claim IP protection (generally on downstream inventions where further research would add ‘intellectual and resource capital…’^164^), such claims should not inhibit data sharing. One consortium document discourages the claiming of IP by members.

In total, then, 20 consortia make some provision for the ownership of IP produced through use of the data. Coupled with the NIH and other data sharing policies discussed above, we can conclude that around half of the consortia examined specifically contemplate the claiming of IP in consortia documentation. In remaining cases, it may be that IP over outputs is either rarely contemplated, or simply not dealt with.

A number of OA consortia make provision for IP over data contributed by consortia members. Fourteen consortia documents make some mention of existing IP attached to data contributed to consortia, with the vast majority stating that this IP would remain the property of the contributing party. In reality, there is little doubt that legally this would be the case, so that such clauses simply restate the law. In any case they are hardly a surprising inclusion. In total, six consortia make reference in their documentation to the status of *both* existing IP, and IP generated through use of consortia data.

Table 3 summarizes these findings.

**Table 3:** IP clauses in consortium agreements

	CA	OA	Registered access	MA	TA	Total
Prohibits claiming of IP over outputs				1	4^a^	5
Discourages claiming of IP over outputs			1		1^b^	1
Permits IP to be claimed over outputs				1	4^c^	5
Unclear/no information					2^d^	2
Claiming IP over outputs may be necessary but should not inhibit sharing of data					5^e^	5
Consortium or funding body owns newly developed IP	1				3^f^	4
Contributors own existing IP connected with contributed data				1	13	14

^a^Includes two NIH-funded consortia.

^b^Is an NIH-funded consortium (CSER).

^c^Includes two NIH-funded consortia.

^d^One is an NIH-funded consortium (PsychEncode).

^e^Three NIH-funded and two Wellcome-funded.

^f^One of these consortia (the Autism Sequencing Consortium) also appeared to permit IP to be claimed by contributors in its guidelines, but encouraged ‘mutual benefit’: ‘All parties to the collaboration benefit. The first and foremost benefit of the ASC collaboration will be finding the genes that confer susceptibility to ASDs, leading to a better understanding of the causes of ASDs and development of potential treatments. The investigators will also benefit from authorship on ASC publications and may also benefit through increased or stable funding, further learning opportunities, the potential for career advancement, and learning more about the most appropriate strategies to uncover the genetic mechanisms of complex disease. The investigators and funding agencies may also benefit from owning intellectual property from any discoveries that are made. There is a clear recognition that for this collaboration to go ahead, any decisions that are made must attempt to provide mutual benefit for all those involved in the ASC, not just those directly responsible for a particular discovery. Admittedly it will be difficult to ensure that the benefit is equally distributed, or that it is equal in kind among all partners; nevertheless there must be an assurance of mutual benefit.’: ASC, Memorandum of Understanding (June 23, 2014), https://genome.emory.edu/ASC/?page_id=131.

Again, markedly more TA consortia include provisions in relation to claiming IP, which again is expected, given that they comprise the greatest number of consortia examined. There is very little spread across consortia types, and on the whole, TA consortia (a majority of which were NIH-affiliated) are more likely to contain some stipulation as to the status of IP contributed to consortia, and to ownership of IP generated through use of consortia data, favoring the granting of IP ownership rights to researchers or users of data. There are very few express prohibitions against claiming IP, indicating that IP is the most accepted form of ‘ownership right’ over the outputs of consortia data, and reflecting some recognition that legally, IP resides in users of consortium data who produce IP-protectable outputs from that data. Thus, the inclusion of *indirect* means of recognizing interests in consortium data (via broad definitions of data and IP in IP-related clauses) can be observed to be the most-favored method of dealing with the uncertainty around ownership of genomic data. The use of IP to navigate this uncertainty is an interesting way of resolving the uncertainty given there remains some doubt around the eligibility of genomic datasets for IP protection, but it indicates that consortia administrators consider such protection to be a surer bet than other forms of ownership.

### V.F. Provisions on Donor Privacy/Protection

Given that strong privacy laws are effectively an alternative to vesting ownership interests in donors of genomic data, we considered the extent to which consortia documentation contained provision for protection of donor privacy, and/or confidentiality. In total, 29 consortia have clearly articulated terms in policy documents making reference to the privacy of donor data.

Of these terms, many are very similar. They generally fall into two categories: those that make some statement requiring data contributors to ensure that contributed data is adequately de-identified, and those that require some commitment from researchers seeking to access consortium data, to not attempt to re-identify data subjects (or at least stated that attempts should not be made to re-identify). Some consortia documents (eight) mention the importance of maintaining privacy for both data contributors *and* data accessors. In some cases, explicit reference is made to relevant laws guiding data practices. The results from this institutional count are presented in Table 4.

**Table 4:** Provisions as to donor privacy/protection against jurisdiction

Jurisdictional origin	Terms relating to data contribution	Terms relating to data access	Terms relating to contribution and access
US	3	10^a^	2
EU	1	3	1
Africa		1	
Australia			1
Asia		1	
Canada		1	
Multi-jurisdictional		1	4
Total	4	17	8

^a^One of these consortia included a term setting out the requirements for maintenance of privacy for those seeking access, and also required that any data breach be notified to data subjects upon it becoming known.

We stress that the absence of specific terms prohibiting the identification or respect for privacy of the participants does not mean that privacy obligations are not conformed with by consortia in question. Often the terms we discovered operate to reiterate or confirm minimum legal obligations that consortia will be required to comply with under jurisdictional laws.^165^ Failure to include such terms does not, therefore, mean that consortia falling into this category were any less likely to maintain high thresholds for donor confidentiality. Indeed, consortia governed by NIH and Wellcome policies are required to adhere to well-articulated guidelines on protection of donor privacy in respect of both contributed data and accessing data.^166^

Whether the legal standards for privacy are adequate is another question, and there are divergences between jurisdictions. Australia, for example, has a complex web of privacy legislation that imposes fewer obligations on those collecting and holding personal information than the European GDPR.^167^ There did not appear to be any significant difference between the inclusion of terms dealing with privacy on the basis of the jurisdiction in which a particular consortium was established.

On the question of access type, one of the consortia that makes explicit reference to confidentiality or privacy of data releases information on an OA basis.^168^ Indeed, some consortia name the need to retain confidentiality as a reason not to release data on an OA basis. For example, Human Heredity and Health in Africa provides in its Data Access and Release Policy:^169^

To protect the rights and privacy of human subjects who participate in the studies, clinical metadata, genomic, or other datasets, or a subset of the clinical and other metadata that may potentially identify the human subjects who donated samples shall not be released in any publicly accessible databases. Clinical data and other fields that may potentially uniquely identify an individual will be carefully reviewed and flagged prior to sharing and releasing to publicly accessible databases.

The remaining consortia that deal explicitly with privacy in some aspect are TA (25),^170^ while three fall into the MA category. In short, clear recognition of the privacy rights of data donors sends a clear signal from at least half the consortia examined, that although donors are able to exercise limited control over the use of data, the conditions of consent under which the data was collected will be respected. Emphasis on contribution of de-identified data further cements the primacy of consent and privacy considerations.

## VI. DISCUSSION

In our analysis of genomic research consortium governance documentation, we observed a wide variety of different types of genomic research consortia, with disparities across many facets of consortium governance. For instance, while some genomic research consortia can be classified as OA, others are closed to all except members. We also saw many variations on access structures between these ends of the continuum.^171^

Our analysis provides some evidence that genomic research consortia are appropriately dealing with the complex confluence of rights and interests in genomic data. There is little evidence, however, that consortia deal with these issues through the language of property and ownership. Rather, they tend to include in their governance arrangements provision for attribution and, in many instances, donor privacy. These particular terms can be seen to take relatively common forms, no doubt highlighting that their inclusion is prompted by the existence of strong privacy laws with firm boundaries, the precedence placed on attribution and acknowledgment by data contributors and their institutions, and the primacy of these concepts as core components of responsible data sharing frameworks.^172^

It is only in the context of use of consortium research that we see the language of ‘property’, in the form of IP. What is perhaps most surprising is the lack of uniformity in approaches to governance of these significant issues relating to the collection, storage, and use of genomic data between consortia.

This lack of standardization in utilization and drafting of terms is not unique to genomic data consortia and has also been reported in relation to biobanks.^173^ In our study, the frequency of use of terms diverged and so too did the composition of various terms, even when they appeared to be used to achieve similar aims. This suggests the use of templates is uncommon, and founders of consortia are not consulting examples of documents from other consortia that are freely available online. In addition, standard-setting bodies such as GA4GH, the NIH, and the Wellcome Trust have all produced pro forma documents, but substantial variance in the use and drafting of governance documents alludes to the fact that calibrating governance documents to accord with those of other consortia is not the norm.

There are likely to be a number of reasons for this. First, the founders of genomics consortia, particularly smaller-scale consortia with managed-access structures and involving fewer participants, may not view the production of detailed governance documents dealing with ownership issues to be a primary concern, particularly given the prevalence of more pressing governance issues, such as those associated with privacy, donor consent, and rewarding data contributors.^174^ This is evidenced by the comparative preponderance of terms dealing with privacy, attribution, and acknowledgment.

Second, there may be a perception among consortium founders that the inclusion of terms as to ownership in governance documents is not especially important because these terms simply reflect the legal position in any case, particularly in relation to IP. Third, the existence of pro forma documents such as those produced by GA4GH could be viewed as imparting default expectations around property interests in genomic data housed in consortia, rendering the need for consortium-specific terms redundant. Finally, there is marked divergence across consortia structures, purposes, and functions. These differences invariably account in part for the individualization of governance documentation, along with access terms within those documents.

On the whole, the evidence gathered demonstrates that the practices of genomic research consortia are broadly consistent with the legal position. Insofar as proprietary interests are concerned, the legal analysis has shown that there will be difficulty in demonstrating that donors have any form of ownership rights in their genomic data. Rather, their interests are generally protected through recognition of their right to privacy and their right not to be identified. The absence of explicit statements in many consortia documents dealing directly with these issues could signal that privacy laws are seen as providing adequate protection for individuals donating genomic data, and hence where such terms are included, they tend to reflect the legal position rather than attempt to impose contractual requirements that go beyond these legal requirements on data contributors and researchers accessing data or provide greater protection through contractual arrangements.^175^ Because the rights of donors are protected so explicitly through the right to privacy, the right to confidentiality, the right not to be re-identified, and informed consent processes, our analysis also revealed limited evidence of statements pointing to provenance of data.

It was more common, however, to see terms requiring attribution or acknowledgment, and even co-authorship in some cases. Such terms ensure that researchers and consortia are appropriately rewarded for their contribution to the data sharing effort, thereby avoiding the need to assert proprietary rights. Admittedly, researchers are likely to have a stronger claim to proprietary interests in genomic data generated from tissue samples than donors, but it was rare to find evidence of terms recognizing such interests. In any case, it is likely that proprietary interests per se would do little to enhance the capacity of researchers to capitalize on their scholarly input. More likely than not, IP interests will be utilized to protect the exploitation and commercialization of genomic data and compilations of data. We found evidence of a number of terms in consortia documents, some permitting researchers to claim IP rights, and others actively discouraging such practices. It is unlikely that such terms would be effective to prevent researchers from making claims over IP they generate should they choose to do so, given the right to claim IP is enshrined in statute. This is despite the fact that these intellectual outputs are produced using primary data generated by others. In any case, relatively few documents outright prohibit the claiming of IP which perhaps reflects this reality.

Lastly, our documentary analysis revealed very little evidence of *consortia making claims* either to proprietary interests or to IP. The premise behind many consortia is facilitation, and so it is hardly surprising that we do not see extensive evidence of assertion of proprietary interests by them. Again, requirements to attribute consortia were commonly included, but generally this was limited to circumstances where consortia did more than simply provide access to data. Perhaps more importantly, there was also little evidence of consortia addressing issues associated with downstream IP developed using consortium data. Setting boundaries and principles around ownership and licensing of IP is likely to be an important indicator of consortium success.^176^ Establishing what constitutes ‘precompetitive’ as opposed to ‘competitive’ activities of consortium is crucial and can assist in ameliorating ownership issues in respect of newly-developed IP.^177^ Issues that may arise if IP is not adequately dealt with as part of the establishment of a consortium include:^178^

- Uncertainty as to the ownership of background IP;
- Uncertainty as to the ownership of consortium-generated IP;
- Lack of clarity as to who may lawfully exploit IP generated during the life of a consortium;
- A risk that IP may not be maintained after the conclusion of a consortium.

## VII. CONCLUSION

Despite the prevalence of the language of ownership and property rights in the context of genomics, the background material presented in this article shows that the law lacks clarity and certainty. While contractual arrangements dealing with proprietary interests might provide a path through this morass, real questions must be asked about whether the language of property is appropriate in all the circumstances. We have seen a noticeable absence of the language of property in our empirical analysis of genomic research consortia governance documents, probably for good reason. The real value in genomic data lies in its aggregation. Extensive databases of genomic data are invaluable reference points for diagnosis of genetic mutations and disease. The notion of ownership is antithetical to the notion of open genomic consortia: instead, such collections may be better viewed as global public goods^179^ or at least public uses of private data.^180^ Consortia supported by public funding efforts are far more likely to adopt governance systems that eschew assertions of proprietary interests, and to promote attribution of data contributors and adherence to privacy law obligations, to promote the interests of data contributors and donors.

We conclude that this is the correct emphasis; consortia founders and members are right in focusing on rewarding data contributors and protecting data donors. Further, an absence of terms purporting to attribute property interests is entirely appropriate and represents the ambiguous legal position. On the other hand, IP claims over the fruits of innovation are more likely to be successful and thus we would expect to see more uniform acceptance of these terms in governance documents. We acknowledge that the inclusion of IP terms does not change fundamental entitlements to claim IP. However, there is benefit for both data contributors and data users in having access to a clearly articulated IP policy prior to data contribution/use. We therefore recommend future inclusion by consortia of clearly drafted IP terms on the basis that whether or not the claiming of IP by data users is encouraged should be clearly ascertainable while also being compliant with principles for responsible sharing of data.^181^

## Supplementary Material

Supplementary_information_table_19082024_lsae024

## Footnotes

1 See, for example, Mahsa Shabani and Luca Marelli, *Reidentifiability of Genomic Data and the GDPR*, 20 Embo Reports, e48316 (2019). https://doi.org/10.15252/embr.201948316.

2 Kathleen Liddell, David A. Simon and Anneke Lucassen, *Patient Data Ownership: Who Owns Your Health,* 8 Journal of Law and The Biosciences lsab023 (2021), 10.1093/jlb/lsab023.

3 Brett Trost, Livia O. Loureiro and Stephen W. Scherer, *Discovery of Genomic Variation across a Generation*, 30 Human Molecular Genetics 30, R174 (2021), 10.1093/hmg/ddab209; Zeeshan Ahmed, Samed Zeeshan, Dinesh Mendhe and XinQi Dong, *Human Gene and Disease Associations for Clinical-Genomics and Precision Medicine Research*, 10 Clinical Translational Medicine, 297 (2020).

4 Ting Wang, Lucinda Antonacci-Fulton, Kerstin Howe, Heather A Lawson, et al. *The Human Pangenome Project: A Global Resource to Map Genomic Diversity.* 604 Nature, 437 (2022).

5 Juli M. Bollinger, Peter D. Zuk, Mary A. Majumder, Erika Versalovic, et al., *What is a Medical Information Commons*, 47 The Journal of Law, Medicine and Ethics, 41, 42 (2019).

6 Jorge L. Contreras and Bartha M. Knoppers, *The Genomic Commons*, 19 Annual Review of Genomics and Human Genetics, 429, 431 (2018).

7 Patricia A. Deverka, Mary A. Majumder, Angela G. Villanueva, Margaret Anderson et al., *Creating a Data Resource: What Will it Take to Build a Medical Information Commons?,* 9 Genomic Medicine, 1 (2017); Bollinger et al.

8 Contreras and Knoppers, *supra* note 6, at 446.

9 Angela G. Villanueva, Robert Cook-Deegan, Barbara A. Koenig, Patricia A. Deverka, et al., *Characterizing the Biomedical Data-Sharing Landscape,* 47 The Journal of Law, Medicine and Ethics, 21 (2019).

10 See, for example, William Meyerson, John Leisman, Fabio C. P. Navarro and Mark Gerstein, Origins and Characterization of Variants Shared between Databases of Somatic and Germline Human Mutations, 21 Bmc Bioinformatics, 227 (2020).

11 Katharina Sielemann, Alenka Hafner and Boas Pucker, *The Reuse of Public Datasets in the Life Sciences: Potential Risks and Rewards*, 8 Peer J, e9954 (2020), 10.7717/peerj.9954

12 EURAT Project Group, Ethical and Legal Aspects of Whole Genome Sequencing, Position Paper on The Release of Raw Genomic Data to Patients and Study Participants, 23–4 (2019).

13
*Id,* at 25–6.

14 Villanueva et al., *supra* note 9.

15
*Id*, at 24.

16 Dianne Nicol, Jane Nielsen and Madeleine Archer, *Data Access Arrangements in Genomic Research Consortia,* in submission, copy on file with the authors.

17
https://www.ga4gh.org (accessed Nov. 20, 2024). See Bartha M. Knoppers, *Framework for Responsible Sharing of Genomic and Health-related Data*, 8 Hugo*,* Article 3 (2014), 10.1186/s11568-014-0003-1; Yann Joly, Bartha M. Knoppers, Edward Dove and Dianne Nicol, *The GA4GH Regulatory and Ethics Work Stream (REWS) at 10*: *An Interdisciplinary, Participative Approach to International Policy Development in Genomics*, in Data Sharing in Health Sciences and Law (Marcelo C. Compagnucci, Timo Minssen, Mark Fenwick, Matteo Aboy and Kathleen Liddell, eds., Springer, 2024) 13–32.

18 See, eg, *Yanner v Eaton* (1999) 201 CLR 351, 365–67.

19 Wesley N. Hohfeld, Some Fundamental Legal Conceptions as Applied in Judicial Reasoning, 23 Yale L.J., 16, (1913); Anthony M**.** Honore, *Ownership*, in Oxford Essays in Jurisprudence, 107 (Anthony G. Guest, ed, Oxford University Press, 1961).

20 See, eg, Jessica L. Roberts, *Progressive Genetic Ownership*, 93 Notre Dame Law Review, 1105, 1106, 1128, 1130 (2018).

21 Recent case law in the US, UK, and Australia includes: *United States v Craft*, 535 U.S. 274 (2002); *Yearworth and others v North Bristol NHS Trust* (2010); QB 1; *Yanner v Eaton* (1999) 201 CLR 351.

22 Jonathan Montgomery, *Data Sharing and the Idea of Ownership*, 23 The New Bioethics, 81, 84 (2017).

23 Wendy Bonython and Bruce Baer Arnold, *Beyond the Corporeal: Extending Propertisation of Body Parts to Derivative Information,* 23 Journal of Law and Medicine, 688, 691(2016).

24
*Bullock v Dodds* (1818) 2 B & Ald 258 at 276; 106 ER 361 at 368, per Abbott J.

25 See, eg, *Kremen v Cohen* 337 F 3d 1024 (9th Cir. 2003); *Boardman v Phipps* 2 AC 46 [1966] and *OBG v Allan* (2008) 1 AC 1; *Federal Commissioner of Taxation v United Aircraft Corp* (1943) 68 CLR 525. See also Liddell et al., *supra* note 2, at 12–6; Katrina M. Wyman, *Property as Intangible Property*, in Oxford Studies in Private Law Theory: Volume I (Paul B Miller and John Oberdiek (eds), Oxford Academic, 2021), 10.1093/oso/9780198851356.003.0004. See further below in relation to IP.

26 See, eg, *Four Point Garage Ltd v Carter* [1985] 3 All ER 12; G*amer’s Motor Centre (Newcastle) Pty Ltd v Natwest Wholesale Australia Pty Ltd* (1987) 163 CLR 236; Gail Pearson, *Constructive Possession and Constructive Delivery in Transfer of Title to Goods*, 26(1) Unsw Law Journal 159 (2003)

27
*Penfolds Wines Pty Ltd v* Elliott (1946) 74 CLR 204, at 226–8.

28 Bonython and Baer Arnold, *supra* note 23, at 698.

29
*Doodeward v Spence* (1908) 8 CLR 406.

30 Jane Kaye, Jessica Bell, Megan Prictor and Megan Munsie, *Trends and Challenges in Biobanking*, in Tensions and Traumas in Health Law, 415, 429 (Ian Freckelton and Kerry Peterson, eds, The Federation Press, 2017); *Re Organ Retention Group Litigation* QB 621, [148] (2005).

31
*R v Kelly* (1999) 2 WLR 384.

32 Bonython and Baer Arnold, *supra* note 23, at 696–7.

33
*Moore v Regents of the University of California* 793 P 2d 479 (1990); *Greenberg v Miami Children’s Hospital Research Institute* 264 F Supp 2d 1–64 (2003); *Washington University v Catalona* 490 F 3d 667 (2007).

34
*Washington University v Catalona* 490 F 3d 667 (2007).

35 Cameron Stewart, Jennifer Fleming and Ian Kerridge, *The Law of Gifts, Conditional Donation and Biobanking*, 21 Journal of Law and Medicine 351 (2013).

36 See, eg, *Yearworth v North Bristol NHS Trust* [2009] 2 All ER 986; *Roblin v Public Trustee (ACT)* [2015] ACTSC 100.

37 See, eg, *Hecht v Superior Court (Kane)* 20 Cal Reporter 2d 275 (1993); *Re Estate of Edwards* [2011] 81 NSWLR 198; *Creswell v AG for the State of Queensland* [2018] QSC 142.

38 Kaye et al., *supra* note 30, at 429.

39 See, eg, Barbara Evans, *Much Ado about Data Ownership,* 25 Harvard Journal of Law and Technology 69 (2011).

40 We refer to the person from whose tissue sample the genomic information was generated as the donor, recognizing that term ‘donor’ connotes ownership: Annie Sorbie, Wifak Gueddana, Graeme Laurie, David Townend, *Examining the power of the social imaginary through competing narratives of data ownership in health research*, 8(2) Journal of Law and the Biosciences 1 (2021). We do not intend to categorically infer ownership through use of this term.

41 See, eg, Alice Lu, Peter Kuhn and Ida Deichaite, *Time for a Change: Considering the Rights of Study Participants to Ownership of Their Personal Research-Grade Genomic Data*, 4 Convergent Science Physical Oncology 046001 (2018) 10.1088/2057-1739/aaf822; Roberts, *supra* note 20; Katherine A. Mikk, Harry A. Sleeper, Eric J Topol, The Pathway to Patient Data Ownership and Better Health, 318(15) Jama 1433 (2018).

42 The British Academy, The Royal Society and techUK, Data Ownership, Rights and Controls: Reaching a Common Understanding, 5 (Seminar Report, 2018), https://www.thebritishacademy.ac.uk/publications/data-ownership-rights-controls-seminar-report/ (accessed Nov. 20, 2024).

43 See, eg, Contreras and Knoppers, *supra* note 6; Richard A. Spinello, *Property Rights in Genetic Information.* 6 Ethics Inf Technol, 29 (2004). For an extensive review of the arguments for and against property in health information see Liddell et al., *supra* note 2, at 35–47.

44 Maurice Cassier, *Private Property, Collective Property, and Public Property in the Age of Genomics*, 54 International Social Science Journal 83 (2002).

45 Michael A. Heller and Rebecca S. Eisenberg, *Can Patents Deter Innovation? The Anticommons in Biomedical Research,* 280 Science, 698 (1998).

46 Jorge Contreras, *Genetic Property*, 105 Georgetown Law Journal 1, 7 (2016); Amy L. McGuire, Jessica Roberts and Barbara J. Evans, *Who Owns the Data in a Medical Information Commons?,* 47 The Journal of Law, Medicine and Ethics 62, 67 (2019).

47 Kendall Powell, *The Broken Promise that Undermines Human Genome Research,* 590 Nature, 198 (2021).

48 See, eg, Marc A. Rodwin, *The Case for Public Ownership of Patient Data,* 302 Jama 86, 87–8 (2009). See also Liddell et al., *supra* note 2, at 40–3.

49 See, eg, Jorge L Contreras, *The False Promise of Health Data Ownership*, 94 New York University Law Review 624 (2019).

50 Montgomery, *supra* note 22.

51 See, for example, Dianne Nicol, Christine Critchley, Rebekah McWhirter and Tess Whitton, *Understanding Public Reactions to Commercialization of Biobanks and Use of Biobank Resources* 162 Social Science And Medicine, 79 (2016)

52 Angela Ballantyne, *How Should We Think about Clinical Data Ownership?*, 46 Journal of Medical Ethics 289, (2020); Barbara Evans, *Barbarians at the Gate: Consumer-Driven Health Data Commons and the Transformation of Citizen Science* 42(4) American Journal of Law and Medicine 652 (2016).

53 Paula Boddington, *Ethical Challenges in Genomics Research: A Guide to Understanding Ethics in Context*, ProQuest Ebook (2012), 211.

54 Evans, *supra* note 46, at 79–81.

55
*Id,* at 79.

56 Contreras, *supra* note 46.

57
*Id.*

58 Evans, *supra* note 46, at 79–81.

59 The power to enact such statutes is contained in s 51(xxxi) of the Constitution of Australia.

60 Evans, *supra* note 46, at 80–1.

61 Heller and Eisenberg, *supra* note 45.

62 Montgomery, *supra* note 22, at 82.

63 Pamela Samuelson, Privacy as Intellectual Property, 52 Stanford Law Journal, 1125, 1138 (2000).

64 Henry T. Greely, *The Control of Genetic Research: Involving the ‘Groups Between*’, 33 Houston Law Review 1397, 1398 (1997).

65 David Jefferson, *Biosociality, Reimagined: A Global Distributive Justice Framework for Ownership of Human Genetic Material* 14 Chicago-Kent Journal of Intellectual Property 357, 374–5 (2015).

66 For a recent discussion see Anya E. R. Prince, *Comprehensive Protection of Genetic Information: One Size Privacy or Property Models May Not Fit All*, 79 Brooklyn Law Review 175, 195–8, (2013); Roberts, *supra* note 20, at 1128. For a more recent discussion see Donna M. Gitter, *Achieving Genetic Data Privacy Through Enforcement of Property Rights,* 57 University of California, Davis Law Review 131 (2023), particularly her *supra* note 6 and 158–9. Gitter also discusses the role of other state laws in providing similar levels of protection. See generally 154–61.

67
*Peerenboom v Perlmutter*, No 2013-CA015257 (Fla Cir Ct Apr 7, 2017). See also Gitter, *Id.* at 162–4.

68
*Cole v Gene by Gene Ltd*, No 1:14-cv-00004-SLG, 2017 US Dist LEXIS 101761 (D Alaska June 30, 2017). See also Gitter, *Id.* at 164–6.

69
*Cole v Gene by Gene Ltd*, No 1:14-cv-00004-SLG, 2017 US Dist LEXIS 101761 (D Alaska June 30, 2017), [8].

70 See particularly Liddell et al., *supra* note 2.

71 Teresa Scassa, *Data Ownership* CIGI PAPERS NO 187, 10, 15 (Centre for International Governance Innovation, 2018).

72 EU General Data Protection Regulation (GDPR): Regulation (EU) 2016/679 of the European Parliament and of the Council of Apr. 27, 2016 on the protection of natural persons with regard to the processing of personal data and on the free movement of such data, and repealing Directive 95/46/EC, OJ 2016 L 119/1.

73 Organisation for Economic Cooperation and Development, Data in the Digital Age, 4 (Mar. 2019).

74 See, eg, Scassa, *supra* note 71.

75 See, eg, Carolyn Johnston, Jane Nielsen, Mark J Cowley, Rebekah McWhirter and Margaret Otlowski, *They Can Have Our Cake—But Can We Eat It? Access to Raw Genomic Data under Australian Privacy Law*, 30 Journal of law and medicine 616 (2023).

76
*Breen v Williams* (1996) 186 CLR 71.

77 Office of the Information Commissioner, Freedom of Information (2020) https://www.oaic.gov.au/privacy/health-information/ (accessed Nov. 20 , 2024).

78
*Breen v Williams* (1996) 186 CLR 71.

79 Health Insurance Portability and Accountability Act (HIPAA) Privacy Rule, https://www.hhs.gov/hipaa/for-professionals/privacy/index.html (accessed Nov. 20 , 2024). See Deverka et al., *supra* note 7.

80 Jonathan L. Mezrich and Eliot Siegel, *Who Owns the Image? Archiving and Retention Issues in the Digital Age,* 11 Journal of the American College of Radiology, 384 (2014).

81 Nadezhda Purtova, *Do Property Rights in Personal Data Make Sense after the Big Data Turn?*, 10 Journal of Law and Economic Regulation 208, 214 (2017).

82
*Breen v Williams* (1996) 186 CLR 71.

83 Liddell et al., *supra* note 2.

84 McGuire et al., *supra* note 46, at 64.

85 Cf Lothar Determann, *No One Owns Data*, 70 Hastings Law Journal 1 (2018).

86 A*ssociation for Molecular Pathology v Myriad Genetics Inc,* 133 US 2107 (2013); *D’Arcy v Myriad Genetics Inc* (2014) 224 FCR 479.

87 A*ssociation for Molecular Pathology v Myriad Genetics Inc,* 133 US 2107 (2013).

88
*Association for Molecular Pathology v Myriad Genetics Inc,* 133 US 2107 (2013), 2119.

89
*D’Arcy v Myriad Genetics Inc* (2014) 224 FCR 479, [91] per the plurality.

90 Convention on the Grant of European Patents, art. 52(3), Oct. 5, 1973, 1065 U.N.T.S. 255, 13 I.L.M. 270; Council Directive 98/44/EC, 1998 O.J. (L 213) 13–4 (EC). Case T 1213/05, Breast and Ovarian Cancer/University of Utah Research Foundation, at ¶¶ 43–5, 46. There is an extensive body of literature on the US and Australian decisions, and on the differences in approaches between the US, Europe, and Australia. The authors and colleagues provide detailed accounts of these matters in: Rochelle Dreyfuss, Jane Nielsen and Dianne Nicol, *Patenting Nature—A Comparative Perspective*, 5 Journal of Law and the Biosciences 550 (2018); Dianne Nicol, Rochelle C. Dreyfuss, E. Richard Gold, Wei Li, John Liddicoat and Geertrui Van Overwalle, *International Divergence in Gene Patenting,* 20 Annual Review of Genomics and Human Genetics 519 (2019).

91 See Dreyfuss et al., *Id*.

92 See the useful overview in Arti Kaur Rai, Colleen V. Chien and Jenna Clark, *Molecular Diagnostics Patenting After Mayo v Prometheus: An Empirical Analysis* (Nov. 29, 2024). Duke Law School Public Law & Legal Theory Series No. 2023-69, Available at SSRN: https://ssrn.com/abstract=4648623 or 10.2139/ssrn.4648623

93 Rai et al., *Id*.

94 Scassa, *supra* note 71.

95
*Primary Health Care Ltd v Commissioner of Taxation* FCA 419 [123]–[141], (2010)

96 Australian Law Reform Commission (ALRC), Genes and Ingenuity: Gene Patenting and Human Health, Report NO 99, [28.24]. (Commonwealth of Australia, 2004).

97 Sue Coke, *Copyright and Gene Technology*, 10 Journal of Law and Medicine 97 (2002) Cf Beatriz San Martin and Heidi Hurdle, *An Alternative to Patents: Can DNA be Protected by Copyright and Design Right Law?,* 3 Cell Gene Therapy Insights, 639 (2017).

98 US Copyright Office, Compendium of Us Copyright Office Practices §313.3(A) (3d ed, 2021).

99 See, eg, Andrew W. Torrance, ‘DNA Copyright’ (2011) 46 Val UL Rev 1; Christopher Holman, Claus Gustafsson and Andrew W Torrance, *Are Engineered Genetic Sequences Copyrightable?: The US Copyright Office Addresses a Matter of First Impression,* 35 Biotechnology L Rep, 103 (2016); Devdatta Malshe, *Copyrighting DNA: An Off-Label Use,* 19 Wake Forest J Bus and Intell Prop Law, 34 (2018).

100 Christopher M. Holman*, Copyright for Engineered DNA (Part 3),*  Gq Life Sciences Blog (Mar. 8, 2016), https://www.lexology.com/library/detail.aspx?g=4f24f8cc-1319-487f-b307-f949f215967d; (last accessed Nov. 20, 2024); Christopher M. Holman, *Charting the Contours of a Copyright Regime Optimized for Engineered Genetic Code,* 69 Oklahoma Law Review, 399 (2017).

101 Nina Srejovic, *Copyright Protection for Works in the Language of Life*, 97 Washington Law Review, 459 (2022).

102
*Feist Publications Inc v Rural Telephone Service Co* 499 US 340, 111 S Ct 1282 (1991).

103
*Ice TV Pty Ltd v Nine Network Australia Pty Ltd* (2009) 239 CLR 458; *Telstra Corporation Ltd v Phone Directories Pty Ltd* (2010) 194 FCR 142.

104
*Geophysical Service Inc v Encana Corp* ABQB 230 [2016]. Leave to appeal denied [2–17] CanLii 80435 (SCC) [115].

105 Directive 96/9/EC of the European Parliament and of the Council of Mar. 11, 1996 on the legal protection of databases.

106 Justin Pulford, Taghreed El Hajj, Tara Tancred et al., *How international research consortia can strengthen organisations’ research systems and promote a conductive environment and culture*, BMJ Global Health 2023; 8e011419. 10.1136/bmjgh-2022-011419

107 Michael Morrison, Miranda Mourby, Heather Gowans, Sarah Coy and Jane Kaye, *Governance of research consortia: challenges of implementing Responsible Research and Innovation within Europe*, Life Sciences, Society and Policy (2020) 16:13. 10.1186.s40504-020-00109-z

108 Maurice Cassier, *Private Property, Collective Property, and Public Property in the Age of Genomics*, 54 International Social Science Journal 83, 93 (2002).

109 Jerome A. Singh and Abdallah S. Daar, *Intra-consortium Data Sharing in Multi-National, Multi-Institutional Genomic Studies: Gaps and Guidance,* 3 Hugo Journal, 11 (2009).

110 Singh and Daar, *supra* note 109, at 13; Dave A. Chokshi, Michael Parker and Dominic P. Kwiatkowski, *Data Sharing and Intellectual Property in a Genomic Epidemiology Network: Policies for Large-Scale Research Collaboration,* 85 Bulletin of the World Health Organization 382 (2006).

111
*Id*, at 12.

112 The Bayh-Dole Patent and Trademark Law Amendments Act of 1980 (Pub. L. 96-517) applies to universities and the Stevenson–Wydler Technology Innovation Act of 1980 (Pub. L. 96-480) applies to federal research laboratories.

113
*Stanford University v Roche Molecular Systems, Inc.*, 563 U.S. 776 (2011).

114
*University of Western Australia v Gray* [2009] FCAFC 116.

115
*Oxford University Innovation Limited v Oxford Nanoimaging Limited* [2022] EWHC 3200 (Pat).

116 Contreras, *supra* note 46, at 43–4.

117
https://www.nlm.nih.gov/NIHbmic/nih_data_sharing_repositories.html

118
https://www.ga4gh.org/our-community/driver-projects/#

119 Mailman, M., Feolo, M., Jin, Y. *et al. The NCBI dbGaP database of genotypes and phenotypes.*  Nat Genet 39, 1181–6 (2007). 10.1038/ng1007-1181.

120 Notable exclusions were the Australian Genomics Health Alliance, Public Population Project in Genomics and Society (P^3^G), Global Alliance for Global Health, Melbourne Genomics Health Alliance, Cancer Genomics Consortium (CGC), Clinical Pharmacogenetics Implementation Consortium (CPIC), Genomic Standards Consortium, Genomics England, the RNAi Consortium, the NIST Genome Editing Consortium, Microarray Quality Control (MAQC) Consortium and Bioshare Accelerating Medicines Partnership European Joint Programme on Rare Diseases (EJP RD).

121 Nicol et al., *supra* note 16; Villanueva et al., *supra* note 9, at 24.

122 Paul R. Burton, Natalie Banner, Mark J. Elliot, Bartha Maria Knoppers, James Banks, *Policies and Strategies to Facilitate Secondary Use of Research Data in the Health Sciences* 46(6) International Journal of Epidemiology, 1729 (2017).

123 Also referred to as ‘closed access’ arrangements: Villanueva et al., *supra* note 9 at 24–5.

124 See, eg, Yann Joly, Edward S. Dove, Bartha M. Knoppers, Martin Bobrow, Don Chalmers, *Data Sharing in the Post-Genomic World: The Experience of the International Cancer Genome Consortium (ICGC) Data Access Compliance Office (DACO)*, 8(7) Plos Computational Biology (2012).

125 Yann Joly. Stephanie O. M. Dyke, Bartha Knoppers, Tomi Pastinen, *Are Data Sharing and Privacy Protection Mutually Exclusive?* 167 Cell 1151 (2016).

126 Also referred to as managed access or controlled access: *Id.*; Katrina Learned, Ann Durbin, Robert Currie, Ellen Towle Kephart, Holly C. Beale et al., *Barriers to Accessing Public Cancer Genomic Data* 6 Nature Scientific Data 98 (2019).

127 Villanueva et al., *supra* note 9, at 23–5.

128 Joly et al., *supra* note 125.

129 Nicol et al., *supra* note 16.

130 In addition to documents available to the public at large, a small number of documents were provided to the research team via email upon request.

131 National Institutes of Health, NIH Genomic Data Sharing Policy (2014) https://grants.nih.gov/grants/guide/notice-files/NOT-OD-14-124.html (accessed Nov. 20, 2024). See also 2023 NIH Data Management and Sharing Policy (2023) https://oir.nih.gov/sourcebook/intramural-program-oversight/intramural-data-sharing/2023-nih-data-management-sharing-policy (accessed Nov. 20, 2024).

132 Wellcome, Grant Funding Policies: https://wellcome.org/grant-funding/guidance/grant-funding-policies (accessed Nov. 20, 2024).

133
*Id*.

134 Matthew Jordan, Johnathon Liddicoat and Kathleen Liddell, *An empirical study of large, human biobanks: intellectual property policies and financial conditions for access*, 8(1) Journal of Law and the Biosciences (2021).

135
*Id*, at 13, Supplementary Material, Table F, H.

136 Below, V.F.

137 James W. Hazel and Christopher Slobogin, *Who Knows What, and When?: A Survey of the Privacy Policies Proffered by US Direct-To-Consumer Genetic Testing Companies,* 28 Cornell Journal of Law and Public Policy 35 (2018).

138 Ellen Wright Clayton, Barbara J. Evans, James W. Hazel and Mark Rothstein, *The Law of Genetic Privacy: Applications, Implications and Limitations*, 6 Journal of Law and The Biosciences 1, 18 (2019).

139 The International Cerebral Palsy Genomics Consortium, CP Commons and Cerebral Palsy Alliance, The CP Commons Launch Report (undated), https://icpgc.org/wp-content/uploads/2021/10/CP-Commons-Launch-Report.pdf (accessed Nov. 20, 2024).

140 Broad Institute, Memorandum of Understanding: Participation in the Genome Aggregation Database (GNOMAD) (undated), https://gnomad.broadinstitute.org/mou (accessed Nov. 20, 2024).

141 Solve-RD, Code of Conduct (2020) https://solve-rd.eu/wp-content/uploads/2021/02/Solve-RD_code-of-conduct_v2_1_20201218_final.pdf (accessed Nov. 20, 2024).

142
*Id*.

143 See, eg, the stipulations in the data sharing policies of the Wellcome Sanger Institute, Open Access Science (undated) https://www.sanger.ac.uk/about/research-policies/open-access-science/ (accessed Nov. 20, 2024). These requirements govern research consortia funded by the Institute, eg, Transforming Genetic Medicine Initiative.

144 See, eg, The International Cerebral Palsy Genomics Consortium, *supra* note 139 Appendix 2, New Project Application Form: Data Upload.

145 TaRGET II Consortium also requires reference to GEO accession numbers of datasets used.

146 See, eg, National Institutes of Health, NIH Genomic Data User Code of Conduct (2019) https://osp.od.nih.gov/wp-content/uploads/Genomic_Data_User_Code_of_Conduct.pdf (accessed Nov. 20, 2024). This code of conduct applies to all NIH-funded research studies and consortia.

147 Gnomad, Policies at https://gnomad.broadinstitute.org/policies (accessed Nov. 20, 2024).

148
Genomics England, The National Genomic Research Library V5.1: Amendment To The National Genomics Research and Healthcare Knowledgebase V5 (Genomics England Limited, 2020) (copy on file with authors).

149 International Human Epigenome Consortium, Goals, Structure, Policies & Guidelines (Sept. 10, 2015), https://ihec-epigenomes.org/about/policies-and-guidelines/ (accessed Nov. 20, 2024).

150 PsychENCODE, NIMH Center For Collaborative Genetic Studies Distribution Agreement, (2022) https://www.nimhgenetics.org/request-access/how-to-request-access (accessed Nov. 20, 2024).

151 ICGC-ARGO, E2, Quality Standards of Data: Guidelines regarding the quality and submission of clinical data.

152 See, eg, The International Cerebral Palsy Genomics Consortium, *supra* note 139, Appendix 1, CP Commons Sign-Up Form, and Appendix 3, New Project Application Form: Data Access.

153 See eg, NIH, *supra* note 131, IVC(2): Investigators should register all studies with human genomic data that fall within the scope of the GDS Policy in dbGaP by the time that data cleaning and quality control measures begin, regardless of which NIH-designated data repository will receive the data. After registration in dbGaP, investigators should submit the data to the relevant NIH-designated data repository (eg dbGaP, GEO, SRA, the Cancer Genomics Hub).

154 See Wellcome, *supra* note 132, cl3: ‘in particular, researchers should make sure their shared outputs: are discoverable; use recognised community repositories for data and other outputs where these exist; use persistent identifiers for these outputs wherever possible.’; cl5: ‘Wellcome expects all users of research data…to cite the source, and to abide by the terms and conditions under which they were accessed’.

155 Thijs Devriendt, Pascal Borry and Mahsa Shabani, *Credit and Recognition for Contributions to Data-Sharing Platforms among Cohort Holders and Platform Developers in Europe: Interview Study*, 24 Journal of Medical Internet Research, e25983 (2022). 10.2196/25983.

156 Robert Cook-Deegan, Rachel A. Ankeny and Kathryn Maxson Jones, *“Sharing Data to Build a Medical Information Commons” from Bermuda to the Global Alliance*, 18 Annual Review of Genomics and Human Genetics, 389–415 (2017).

157
https://www.sanger.ac.uk/about/research-policies/open-access-science/ (accessed Nov. 20, 2024).

158 NIH, *supra* note 131. See also 2023 National Institutes of Health, *Data Management and Sharing Policy* (2023) https://oir.nih.gov/sourcebook/intramural-program-oversight/intramural-data-sharing/2023-nih-data-management-sharing-policy (accessed Nov. 20, 2024).

159 See *supra* note 154.

160 Jordan, Liddicoat and Liddell, *supra* note 134, 13.

161 The Alliance of Genome Resources Portal states that all annotations and data produced by Alliance members are distributed under a creative commons licence, but different licensing and copyright terms may apply for data provided by these resources that are not distributed by the Alliance.

162 The eMERGE consortium does so through adoption of the NHGRI Copyright Policy which requires citation of the original copyright owner.

163 H3Africa (patenting encouraged if leads to private investment); the Structural Genomics Consortium (SGC scientists must not file patent applications); Common Mind (there may be patent rights over biomaterials, and no licenses are provided or warranties issued that biomaterials, clinical data, and genetic analysis data may be exploited without infringing the intellectual property or proprietary rights of third parties); Type 1 Diabetes Genetics Consortium (consortium will not claim patents and will not assert IP claims; individual investigators may generate patentable information or material).

164 ICGC-Argo, Intellectual Property Policy https://www.icgc-argo.org/page/78/e4-intellectual-property (accessed Nov. 20, 2024).

165 Privacy laws across the jurisdictions in which these consortia operate are complex and multi-faceted, and it is not proposed that they be considered in detail in this paper. For elaboration on the privacy laws in the various jurisdictions considered, see, eg, Jorge Contreras and Bartha M. Knoppers, *The Genomic Commons,* 19 Annual Rev Genom Hum Genet, 429 (2018).

166 NIH, *supra* note 131; Wellcome, *supra* note 132.

167 Although note that a recent review of Australia’s privacy laws has recommended significant changes that would bring Australian law in line with the standards laid down in the GDPR: Attorney-General’s Department, Privacy Act Review Report 2022 (Commonwealth of Australia, 2022), https://www.ag.gov.au/rights-and-protections/publications/privacy-act-review-report (accessed Nov. 20, 2024).

168 Structural Genomics Consortium, Open Science Policy at https://www.thesgc.org/about/open-science (accessed Nov. 20, 2024).

169 H3Africa, Consortium Data Sharing, Access and Release Policy (Apr. 2020), https://h3africa.org/wp-content/uploads/2020/06/H3Africa-Consortium-Data-Access-Release-Policy-April-2020.pdf (accessed Nov. 20, 2024).

170 Note that one of these consortia (CanPath) also releases data on an open access basis in some instances.

171 See also Nicol et al., *supra* note 16.

172 GA4GH, Framework for responsible sharing of genomic and health-related data (v1.0) (Sept. 3, 2019). See also World Health Organisation, Draft WHO principles for human genome data access, use and sharing (Apr. 8, 2024).

173 Jordan, Liddicoat, and Liddell, *supra* note 134, at 21–3.

174 See also *Id.* at 21–2.

175 Contreras, *supra* note 46.

176
FasterCures, Consortia-Pedia: An In-Depth Look at the Research-By-Consortium Trend in Medical Research and Development, 16–9 (Milken Institute, 2013), https://milkeninstitute.org/sites/default/files/reports-pdf/45700-ConsortiaReport_0.pdf (accessed Nov. 20, 2024).

177
*Id*.

178
*Id*.

179 Ruth Chadwick and Sarah Wilson, *Genomic Databases as Global Public Goods?* 10 Res Publica, 123 (2004).

180 Evans, *supra* note 46, at 119–28.

181 GA4GH, *supra* note 179; WHO, *supra* note 179.

