# Supplementary material for: Data ownership in genomic research consortia
Source: J Law Biosci. 2024 Dec 6;11(2):lsae024. doi: 10.1093/jlb/lsae024 (PMC11630733; doi:10.1093/jlb/lsae024)
Supplement: Supplementary_information_table_19082024_lsae024 [file supplementary_information_table_19082024_lsae024.docx]

| **Supplementary information:  List of consortia in the dataset, including acronyms, consortium types, access types and other demographics** | | | | | | | | |
| --- | --- | --- | --- | --- | --- | --- | --- | --- |
|  |  |  |  |  |  |  |  |  |
| **Consortium** | **Acronym** | **Consortium type** | **Access Type** | **Funding and other affiliations^** | **Date of formation#** | **Administering country/ region** | **Primary source of data on access type*** | **Website** |
| 100,000 Genomes Project |  | National initiative | MA | NHS England; Genomics England; Wellcome | 2012 | UK | Website | <https://www.genomicsengland.co.uk/initiatives/100000-genomes-project> |
| 1000 Genomes Project (the International Genome Sample Resource) | IGSR | Global initiative | TA (CA-OA-MA) | NIH; Wellcome | 2008 | EU | Website | <https://www.internationalgenome.org/home> |
| African American Breast Cancer Epidemiology and Risk Consortium | AMBER | Disease specific – breast cancer | TA (CA-OA-MA) | NIH | 2012 | US | Inferred (repository) | <https://www.ncbi.nlm.nih.gov/projects/gap/cgi-bin/study.cgi?study_id=phs000669.v1.p1>  (Amber Consortium website is no longer live) |
| All of Us | All of Us | National initiative | TA (CA-OA-MA) | NIH | 2015 | US | Website | <https://allofus.nih.gov> |
| Alzheimer's Disease Genetics Consortium | ADGC | Disease specific – Alzheimer’s | TA (CA-OA-MA) | NIH | 2011 | US | Inferred (funders) | <https://www.adgenetics.org> |
| American Association for Cancer Research Project Genomics Evidence Neoplasia Information Exchange | AACR GENIE | General - cancer | TA (CA-OA-MA) | American Association for Cancer Research |  | US | AACR Project Genie et al, 2017 | <https://www.aacr.org/professionals/research/aacr-project-genie/> |
| Athlome Project Consortium |  | General – athletic performance and injury | TA (CA-OA) | Multiple | 2015 | Multinational | Website | <https://www.athlomeconsortium.org> |
| Atrial Fibrillation Genetics Consortium | AFGen | Disease specific – atrial fibrillation | TA (CA-OA-MA) | NIH and others | Pre-2009 | Multinational | Inferred (funders) | <https://www.afgen.org> |
| Autism Genome Project | AGP | Disease specific - autism | TA (CA-OA-MA) | NIH | 2005 | US | Inferred (funders) | <https://www.ncbi.nlm.nih.gov/projects/gap/cgi-bin/study.cgi?study_id=phs000267.v1.p1>  (no specific website) |
| Autism Sequencing Consortium | ASC | Disease specific - autism | TA (CA-OA-MA) | NIH and others | 20i10 | US | *Data Use Certification Agreement*, funders | <https://asc.broadinstitute.org> |
| Autism Sharing Initiative | ASI | Disease specific - autism | MA | Multiple | 2019 | Canada | Websites | <https://www.autismsharinginitiative.org>  <https://www.digitalsupercluster.ca/projects/autism-sharing-initiative/> |
| BLUEPRINT | BLUEPRINT | General – blood diseases | TA (OA-MA) | NIH, Wellcome and others | 2011 | Europe | Website, *Data Access Guidance* | <https://projects.ensembl.org/blueprint/> |
| BRAIN Initiative Cell Census Network Consortium | BICCN | General - brain | TA (CA-OA-MA) | NIH | 2013 | US | Website | <https://www.biccn.org> |
| Brazilian Initiative on Precision Medicine | BiPMed | National initiative | TA (OA-MA) | São Paulo Research Foundation | 2015 | Brazil | Inferred (website and documents) | <https://www.bipmed.org> |
| Breast Cancer Association Consortium | BCAC | Disease specific – breast cancer | TA (CA-MA) | Cancer Research UK, European Commission | 2005 | UK | *Data Access Statement* | <https://www.dkfz.de/en/epidemiologie-krebserkrankungen/units/genepi/ge_pr03_BCAC.html>  (no specific website) |
| Canadian Partnership for Tomorrow Project/Canadian Partnership for Tomorrow’s Health | CanPath | National initiative | MA | National and provincial governments | 2008 | Canada | *Access Policy* | <https://canpath.ca> |
| Cancer Breakthroughs 2020 (Cancer Moonshot, Cancer Moonshot 2020) |  | Broad - cancer | TA (CA-OA-MA) | NIH | 2016/2020 | US | Inferred (funders, Singer 2022) | <https://www.cancer.gov/research/key-initiatives/moonshot-cancer-initiative> |
| Cancer Core Europe | CCE | Broad - cancer | CA |  | 2014 | EU | Inferred (website and documents) | <https://www.cancercoreeurope.eu> |
| Cancer Genome Atlas | TCGA | Broad - cancers | TA (OA-MA) | NIH | 2006 | US | Website | <https://www.cancer.gov/ccg/research/genome-sequencing/tcga> |
| Cancer Genomics of the Kidney | Cage Kid | Broad – kidney cancer | TA (RA-MA) | EU Framework Program; ICGC | 2010 | EU | Inferred (part of ICGC) | <https://cordis.europa.eu/project/id/241669/it>  (CAGE KID website no longer active) |
| Centers for Mendelian Genomics | CMG | Broad – Mendelian disease | TA (CA-OA-MA) | NIH | Pre-2014 | US | Baxter et al 2022 | <https://www.genome.gov/Funded-Programs-Projects/NHGRI-Genome-Sequencing-Program/Centers-for-Mendelian-Genomics-CMG> |
| Clinical Sequencing Evidence-Generating Research | CSER | National Initiative | TA (CA-OA-MA) | NIH | 2011 | US | Inferred (*Principles and Procedures for Consortium of Population-Based Cohort Studies*; NHGRI website) | <https://anvilproject.org/consortia/cser>  <https://www.genome.gov/Funded-Programs-Projects/Clinical-Sequencing-Evidence-Generating-Research-CSER2> |
| Cohorts for Heart and Aging Research in Genomic Epidemiology Consortium | CHARGE | Broad – heart and aging | TA (CA-OA-MA) | NIH | 2008 | US | Inferred (funders; Psaty et al 2009) | <https://www.hgsc.bcm.edu/human/charge-consortium> |
| CommonMind Consortium | CMC | Disease specific - Schizophrenia and Bipolar Disorder | TA (CA-OA-MA) | NIH | 2019 | US | Inferred (synapse platform) | <https://www.synapse.org> |
| Consortium for the Study of Chronic Pancreatitis, Diabetes, and Pancreatic Cancer | CPDPC | Disease specific – pancreatic diseases and cancers | TA (CA-OA-MA) | NIH | 2015 | US | Inferred (funders) | <https://www.cpdpc-research-consortium.org> |
| Consortium of Investigators of Modifiers of BRCA1/2 | CIMBA | Disease specific – breast cancer | MA | Multiple, including Cancer Research UK; NIH | 2006 | UK | *Consortium Application Form* | <https://www.ncbi.nlm.nih.gov/projects/gap/cgi-bin/study.cgi?study_id=phs001321.v1.p1>  (consortium website not active) |
| Critical Assessment of Genome Interpretation | CAGI | National initiative | TA (CA-OA-MA) | NIH | 2010 | US | *Data Use Agreement* | <https://genomeinterpretation.org> |
| Diabetes Research on Patient Stratification | DIRECT | Disease specific - diabetes | TA (CA-MA) | EU Innovative Medicines Initiative | 2012 | UK | *Data Access Policy* | <https://directdiabetes.org> |
| Electronic Medical Records and Genomics | eMERGE | National initiative | TA (CA-OA-MA) | NIH | 2007 | US | *Data Use Agreement* | <https://www.genome.gov/Funded-Programs-Projects/eMERGE-Genomics-Risk-Assessment-and-Management-Network> |
| Encyclopedia of DNA elements | ENCODE | National initiative | TA (OA-MA) | NIH; IHEC | 2003 | US | *Data Use, Software Analysis and Release Policy* | <https://www.encodeproject.org> |
| Endometrial Cancer Association Consortium | ECAC | Disease specific – endometrial cancer | TA (CA-OA-MA) | Australian NHMRC; NIH | 2011 | Australia | Inferred (funders) | <https://ecac-studies.org> |
| European Canadian Cancer Network | EUCANCAN | General - cancers | MA | EU Horizon 2020, CIHR | 2019 | EU, Canada | Website | <https://eucancan.com> |
| European Network for Genetic and Genomic Epidemiology | ENGAGE | Regional initiative | TA (CA-OA-MA) | EU | 2008 | EU | *Principles for Data Sharing, Data Release and Intellectual Property* | <https://www.engage-eu.eu/consortium> |
| Evidence-based Network for the Interpretation of Germline Mutant Alleles | ENIGMA | Disease specific – breast cancer | RA | NIH and others | 2009 | US | Website: Frequently asked questions about ENIGMA | <https://enigmaconsortium.org> |
| Exome Aggregation Consortium | ExAC | National initiative | TA (CA-OA) | Broad Institute; NIH | 2017 | US | Inferred (funders) | <https://ngdc.cncb.ac.cn/databasecommons/database/id/3774> |
| Functional Annotation of the Mammalian Genome | FANTOM | Global initiative | TA (CA-OA) | RIKEN | 2000 | Japan | Kawaji et al 2017 | <https://fantom.gsc.riken.jp> |
| Gene Quality of Life Consortium | GeneQol | Global initiative | TA (CA-MA) | NIH and others | 2009 | The Netherlands | Sprangers et al 2009 | No current website |
| Genetics of Endophenotypes of Neurofunction to Understand Schizophrenia Consortium | GENUS | Disease specific- schizophrenia | CA | NIH and others | Pre-2018 | US | Inferred (Blokland et al 2018) | No current website |
| Genome Asia 100K | GAsP | Regional initiative | TA (CA-MA) | Multiple | 2016 | Singapore | *Data Access Agreement;* Genome Asia100K Consortium 2019 | <https://www.genomeasia100k.org> |
| Genome in a Bottle Consortium | GIAB | National initiative | TA (CA-OA) | NIST – public/private | 2012 | US | Website | <https://www.nist.gov/programs-projects/genome-bottle> |
| Genomics for Understanding Rare Diseases: India Alliance Network | GUaRDIAN | Broad – rare disease | CA | Indian Council of Scientific and Industrial Research | Pre-2019 | India | Inferred (website) | <https://guardian.genomes.in> |
| Genomics Research and Innovation Network | GRIN | National initiative | TA (CA-MA) | NIH and others | 2015 | US | Mandi et al 2020 | No consortium website |
| German Cancer Consortium | DKTK | General - cancer | CA | Federal and state governments | 2012 | Germany | Inferred (website) | <https://dktk.dkfz.de/en> |
| Glioma Gene Consortium | GLIOGENE | Disease specific - glioma | TA (CA-OA-MA) | NIH, National Brain Tumor Society, others | 2007 | US | Inferred (funders) | <https://med.stanford.edu/bondylab/projects/gliogene-project.html/> |
| Glioma Longitudinal Analysis Consortium | GLASS | Disease specific - glioma | TA(CA-RA) | Multiple | 2015 | US | Website | <https://glass-consortium.org>  <https://www.synapse.org/Synapse:syn17038081/wiki/585622> |
| Haplotype Reference Consortium | HRC | Global initiative | OA. | Wellcome | Pre-2016 | UK | McCarthy et al 2016 | <https://www.sanger.ac.uk/collaboration/haplotype-reference-consortium/> |
| Human Genome Project | HGP | Global initiative | OA | Multiple, including NIH and Wellcome | 1990 | Multiple | Bermuda Principles | <https://www.genome.gov/human-genome-project> |
| Human Heredity and Health in Africa | H3Africa | Regional initiative | TA (CA-MA) | NIH; Wellcome | 2010 | South Africa | *Consortium Data Sharing, Access and Release Policy* | <https://h3africa.org> |
| Human Variome Project Consortium | HVP | Global initiative | TA (OA-MA) | Multiple | 2006 | Australia | Ekong and Vihinen 2019 | No current website |
| Implementing Genomics in Practice | IGNITE | National initiative | TA (CA-OA-MA) | NIH | 2013 | US | Website, funders | <https://gmkb.org/ignite-gdp/> |
| International Cancer Genome Consortium Accelerating Research in Genomic Oncology | ICGC ARGO | Global initiative | TA (CA-MA) | Multiple | 2019 | Scotland | *Data Management Policy* | <https://www.icgc-argo.org/> |
| International Cancer Genome Consortium | ICGC | Global initiative | TA (RA-MA) | Multiple | 2007 | Scotland | *Goals, Structures, Policies and Guidelines* | No current website |
| International Cerebral Palsy Genetics Consortium | ICPGC | Disease-specific - cerebral palsy | MA | Multiple | 2017 | Australia | Website | <https://icpgc.org/> |
| International Genomics of Alzheimer's Project | IGAP | Disease-specific - Alzheimer’s | TA (CA-OA-MA) | NIH and others | 2011 | US | Inferred (funders) | No current website |
| International Genomics of Blood Pressure | iGEN-BP | Broad – blood pressure | TA (CA-OA-MA) | NIH and others | Pre-2011 | US | Inferred (repository) | <https://www.ncbi.nlm.nih.gov/projects/gap/cgi-bin/study.cgi?study_id=phs000585.v1.p1>  No current website |
| International Haplotype Mapping Project | HapMap | Global initiative | TA (RA-OA) | NIH; Wellcome and others | 2002 | Multiple | Cook Deegan et al 2017 | <https://www.genome.gov/10001688/international-hapmap-project>  No current website |
| International Human Epigenome Consortium | IHEC | Global initiative | TA (OA-MA) | NIH and others | 2010 | US | *Goals, Structure, Policy and Guidelines* | <https://ihec-epigenomes.org/index.html> |
| International Human Microbiome Consortium | IHMC | Global initiative | TA (OA-MA) | NIH and others | 2008 | Multiple | Inferred (Turnbaugh et al 2007) | No current website |
| International hundredK + cohorts consortium | IHCC | Global initiative | TA (CA-OA-MA) | NIH; Wellcome and others | 2018 | Multiple | *Core Data Sharing Principles* | <https://globalgenomics.org/ihcc/overview/> |
| International Inflammatory Bowel Disease Genetics Consortium | IIBDGC | Disease-specific – inflammatory bowel disease | TA (CA-RA) | NIH and others | 1997 | Multiple | Website | <https://www.ibdgenetics.org/> |
| International Multiple Sclerosis Genetics Consortium | IMSGC | Disease-specific – multiple sclerosis | TA (RA-MA) | NIH, Wellcome and others | Pre-2005 | Multiple | Website | <https://imsgc.net/> |
| International Parkinson's Disease Genomics Consortium | IPDGC | Disease-specific – Parkinson’s | TA (CA-MA) | NIH, Wellcome and others | 2009 | Multiple | Inferred (website) | <https://pdgenetics.org/> |
| International Rare Diseases Research Consortium | IRDiRC | Broad – rare disease | TA (OA-MA) | NIH; European Commission | 2011 | US, EU | Website, publications (Lochmuller et al, 2017) | <https://irdirc.org/> |
| International Serious Adverse Event Consortium | iSAEC | Broad – drug related serious adverse events | TA (OA-MA) | FDA; Wellcome Trust | 2007 | US | Website | <https://saeconsortium.org/> |
| International Stroke Genetics Consortium | ISGC | Disease-specific - stroke | TA (CA-OA-MA) | NIH | 2007 | US | Website | <https://www.strokegenetics.org/> |
| Kidney-Oriented Understanding of Correcting Ciliopathies Consortium | KOUNCIL | Disease-specific - nephronophthisis | CA | Dutch Kidney Foundation |  | NL | Inferred (website; Renkema et al, 2018) | http://www.kouncil.nl/eng/ |
| Lung Genomics Research Consortium | LGRC | Broad – lung disease | TA (CA-OA-MA) | NIH | 2009 | US | Website; dbGaP | <https://www.lung-genomics.org/>  https://www.ncbi.nlm.nih.gov/projects/gap/cgi-bin/study.cgi?study_id=phs000624.v1.p1 |
| Malaria Genomic Epidemiology Network | MalariaGEN | Disease-specific - malaria | TA (CA-MA) | Wellcome; Grand Challenges in Global Health Initiative; Gates Foundation | 2005 | UK | Websites | <https://www.malariagen.net/> |
| Mayo Genome Consortia | MayoGC | National initiative | CA | Mayo |  | US | Inferred (Bielinski et al, 2011) |  |
| MEGASTROKE Consortium | MEGASTROKE | Disease-specific - stroke | TA (CA-OA-MA) | NIH | 2018 | US, EU | Inferred (Malik et al, 2018) | https://www.megastroke.org |
| Molecular Taxonomy of Breast Cancer International Consortium | METABRIC | Disease-specific – breast cancer | TA (CA-MA) | European Genome/Phenome Archive | 2008 | EU | Website | <https://ega-archive.org/studies/EGAS00000000083> |
| MyelomA Genetics International Consortium | MAGIC | Disease-specific – multiple myeloma | CA |  | 2012 | UK | Inferred (Morgan et al, 2012) |  |
| National Ophthalmic Disease Genotyping and Phenotyping Network | eyeGENE* | Broad – inherited eye disease | TA (CA-OA-MA) | NIH | 2006 | US | Website | <https://eyegene.nih.gov/> |
| NIH Common Fund/Roadmap Epigenomics Program |  |  | TA (CA-OA-MA) | NIH | 2008 | US | Website | <https://commonfund.nih.gov/epigenomics> |
| OncoArray Consortium |  |  | TA (CA-OA-MA) | NIH, Genome Canada, Cancer Research UK, EU FP7 | 2013 | US | Website; Amos et al, 2017 | <https://epi.grants.cancer.gov/gameon/>  <https://www.ncbi.nlm.nih.gov/pmc/articles/PMC5224974/> |
| Ovarian Cancer Association Consortium | OCAC | Disease specific – ovarian cancer | TA (CA-MA) |  | 2005 | US | Inferred (Cannioto et al 2017) | https://www.ncbi.nlm.nih.gov/pmc/articles/PMC5616164/pdf/nihms908136.pdf |
| Parkinson's Disease Biomarkers Program | PDBP | Disease specific –  Parkinson’s | TA (CA-OA-MA) | NIH | 2012 | US | Website; Gwinn et al 2017 | <https://pdbp.ninds.nih.gov/data-management-resource>  <https://www.ncbi.nlm.nih.gov/pmc/articles/PMC5619098/> |
| Pediatric & SDH-Deficient Consortium | GIST | Broad - pediatric | TA (CA-OA-MA) |  | 2018 | UK/US | Inferred (website) | <https://liferaftgroup.org/pediatric-sdh-deficient-gist-consortium/> |
| Population Architecture Using Genomics and Epidemiology Consortium | PAGE | National initiative | TA (CA-OA-MA) | NIH | 2008 | US | Website; Matise et al, 2011 | <https://www.pagestudy.org/>  https://pgc.unc.edu/about-us/ |
| Prostate Cancer Association Group to Investigate Cancer Associated Alterations in the Genome | PRACTICAL | Disease specific – prostate | TA (CA-OA) | Cancer Research UK; NCI; EU FP7; Genome Canada | 2008 | UK | Website | <http://practical.icr.ac.uk/blog/> |
| Psych Encyclopedia of DNA elements Consortium | PsychENCODE | Broad - psychiatric | TA (CA-OA-MA) | NIH | 2015 | US | Website | <https://www.psychencode.org/home> |
| Psychiatric Genomics Consortium | PGC | Broad – psychiatric | TA (CA-OA-MA) | NIH | 2007 | US | Website | <https://pgc.unc.edu/about-us/> |
| Rheumatoid Arthritis Map Consortium | RA-MAP | Disease specific – rheumatoid arthritis | TA (CA-MA) | UK Medical Research Council; Various industry partners | 2012 | UK | Website; Cope et al 2018; the RA-MAP Consortium 2022 | <https://research.ncl.ac.uk/ra-map/>  <https://pubmed.ncbi.nlm.nih.gov/35534493/> |
| Scottish Genomes Partnership | SGP | National initiative | TA (OA-MA) | Medical Research Council; Genomics England; NHS | 2015 | UK | Inferred (website) | <https://www.scottishgenomespartnership.org/> |
| Single Nucleotide Polymorphism Consortium | SNP | Global initiative | OA |  | 1999 | US | Thorisson and Stein, 2003 |  |
| Solving the Unsolved Rare Diseases | Solve-RD | Broad – rare diseases | TA (CA-MA) | EU | 2018 | EU | Website | <https://solve-rd.eu/> |
| Structural Genomics Consortium | SGC | Global initiative | OA | NIH; Wellcome; Genome Canada; Gates | 2003 | UK, head office in Canada | Website | <https://www.thesgc.org/> |
| TBResist Global Genome Consortium |  | Disease specific - TB | TA (CA-OA-MA) | NIH; Broad Institute | 2016 | US | Inferred (website, funder) | <https://projects.iq.harvard.edu/tbresist/> |
| The Genetics of Type 2 Diabetes Consortium | GoT2D | Disease specific – diabetes | TA (CA-OA-MA) | NIH; Broad Institute; Wellcome | 2016 | US | Inferred (website, funder) | <https://kp4cd.org/got2d> |
| Toxicant Exposures and Responses by Genomic and Epigenomic Regulators of Transcription Consortium | TaRGET II | Broad - toxicants | TA (CA-OA-MA) | NIH | 2016 | US | Website; *Data Release Policy* | <https://targetepigenomics.org/about/>  <https://targetepigenomics.org/documents/> |
| Transforming Genomic Medicine Initiative | TGMI | Global initative | TA (OA-MA) | Wellcome; Genomics England; NHS; Broad Institute | 2019 | UK | Inferred (website; Wright et al, 2019) | <http://www.thetgmi.org/> |
| Treehouse Childhood Cancer Initiative | TCCI | Broad - cancer | OA |  | 2016 | US | Website; Learned et al 2019 | <https://treehousegenomics.ucsc.edu/> |
| Type 1 Diabetes Genetics Consortium | T1DGC | Disease specific - diabetes | TA (CA-OA-MA) | NIH | 2004 | US | Website | <https://repository.niddk.nih.gov/studies/t1dgc/> |
| Ubiquitous Pharmacogenomics consortium | U-PGx | Broad - pharmacogenomics | MA | EU Horizon 2020 | 2016 | Netherlands | *Ubiquitous Pharmacogenomics Data Management Plan* (2016); Blagec et al, 2018; van der Wouden, 2017 | <https://upgx.eu/> |
| UK10K | UK10K | National initiative | TA (CA-OA-MA) | Wellcome | 2010 | UK | Website; Muddyman et al, 2013; Geihs et al, 2015 | <https://www.sanger.ac.uk/collaboration/uk10k-project/> |
| Variant Interpretation for Cancer Consortium | VICC | Broad - cancer | TA (OA-RA-MA) |  | 2016 | US | *Principles and Policies* | <https://cancervariants.org/principles/> |
| Wellcome Trust Case Control Consortium | WTCCC | National initiative | MA | Wellcome | 2005 | UK | Website | <https://www.wtccc.org.uk/m> |
| Whole Genome Sequencing in Psychiatric Disorders Consortium | WGSPD | Broad – psychiatric disorders | TA (CA-OA-MA) | NIH, Broad Institute | 2017 | US | Sanders et al 2017; funder | <https://www.ncbi.nlm.nih.gov/projects/gap/cgi-bin/study.cgi?study_id=phs002041.v1.p1> (no specific website) |

**Publications cited in the table**

AACR Project Genie Consortium, AACR Project GENIE Consortium, André F, Arnedos M, Baras AS, Baselga J. et al (2017) AACR Project GENIE: powering precision medicine through an international consortium. Cancer Discovery, 7(8):818-831.

Amos CI, Dennis J, Wang A, et al (2017) The Oncoarray Consortium: a network for understanding the genetic architecture of common cancers. Cancer Epidemiol Biomarners Prev, 26(1):126-135

Baxter SM, Posey JE, Lake NJ, Sobreira N, Chong JX, Buyske S, et al (2022) Centers for Mendelian Genomics: A decade of facilitating gene discovery. Genetics in Medicine 24: 784-797

Blages K, Koopmann R, Crommentuijn-vanRhenen M, et al (2018) Implementing pharmacogenomics decision support across seven European countries: The Ubiquitous Pharmacogenomics (U-PGx) project. Journal of the American Medical Informatics Association, 25(7):893-989

Blokland GAM, Del Re EC, Mesholam-Gately RI, Jovicich J, Trampush JW, Keshavan MS,  et al (2018) The Genetics of Endophenotypes of Neurofunction to Understand Schizophrenia (GENUS) consortium: A collaborative cognitive and neuroimaging genetics project. Schizophr Res. 195:306-317.

Cannioto RA, Trabert B, Poole EM and Schildkraut JM (2017) Ovarian cancer epidemiology in the era of collaborative team science. Cancer Causes Control, 28(5):487-495

Cook-Deegan R, Ankeny RA, Maxson Jones K (2017) Sharing data to build a medical information commons: From Bermuda to the Global Alliance. Annu Rev Genomics Hum Genet 18:389-415

Cope AP, Barnes MR, Belson A, et al (2018) The RA-MAP Consortium: a working model for academia-industry collaboration. Nature Rev Rheumatol, 14(1):53-60

Ekong R and Vihinen M (2019) Checklist for gene/disease-specific variation database curators to enable ethical data management. Human Mutation 40:1634-1640

Geihs M, Yan Y, Walter K, et al (2015) An interactive genome browser of association results from the UK10K cohorts project. Bioinformatics, 31(24): 4028-4031

GenomeAsia100K Consortium (2019) The GenomeAsia 100K Project enables genetic discoveries across Asia. Nature, 576:106–111

Gwinn K, David KK, Swanson-Fischer C, et al (2017) Parkinson’s disease biomarkers: perspective from the NINDS Parkinson’s Disease Biomarkers Program. Biomark Med, 11(6):451-473

Kawaji H, Kasukawa T, Forrest A, et al (2017) The FANTOM5 collection, a data series underpinning mammalian transcriptome atlases in diverse cell types. Sci Data 4:170113

Mandl KD, Glauser T, Krantz ID,  Avillach P, Bartels A, Beggs AH, et al*.*(2020) The Genomics Research and Innovation Network: creating an interoperable, federated, genomics learning system. Genet Med 22:371–380

Matise TC, Ambite JL, Buyske S, et al (2011) The next PAGE in understanding complex traits: design for the analysis of population architecture using genetics and epidemiology (PAGE) study. Am J Epidemiol, 174(7):849-859

McCarthy S, Das S, Kretzschmar W, et al (2016) A reference panel of 64,976 haplotypes for genotype imputation. Nature Genetics, 48(10):1279-1283

Muddyman D, Smee C, Griffin H, Kaye J, UK10K Project (2013) The UK10K Project. Implementing a successful data-management framework: the UK10K managed access model. Genome Med 5:100. <https://doi.org/10.1186/gm504>

Psaty BM, O'Donnell CJ, Gudnason V, Lunetta KL, Folsom AR, Rotter JI, et al (2009) Cohorts for Heart and Aging Research in Genomic Epidemiology (CHARGE) Consortium: Design of prospective meta-analyses of genome-wide association studies from five cohorts. Circ Cardiovasc Genet, 2:73-80.

Sanders SJ, Neale BM, Huang H, et al (2017) Whole genome sequencing in psychiatric disorders: the WGSPD consortium. Nature Neuroscience, 20:1661-1668

Singer DS (2022) A new phase of the Cancer Moonshot to end cancer as we know it. Nat Med, 28:1345–1347

The RA-MAP Consortium (2022) RA-MAP, molecular immunological landscapes in early rheumatoid arthritis and healthy vaccine recipients. Sci Data, 9(1):196

Sprangers MA, Sloan JA, Veenhoven R, Cleeland CS, Halyard MY, Abertnethy AP, et al (2009) The establishment of the GENEQOL consortium to investigate the genetic disposition of patient-reported quality-of-life outcomes. Twin Res Hum Genet, 12(3):301-11.

Thorisson GA and Stein LD (2003) The SNP Consortium website: past, present and future. Nucleic Acid Research 31(1):124-127

Turnbaugh P, Ley R, Hamady M, Fraser-Liggett CM, Knight R and Gordon JI (2007) The Human Microbiome Project. Nature 449:804–810

Wright CF, Ware JS, Lucassen AM (2019) Genomic variant sharing: a position statement. Wellcome Open Res, 4: 22

Learned K, Durbin A, Currie C, et al (2019) Barriers to accessing public cancer genomic data. Scientific Data, 6:98

van der Worden CH, Cambon-Thomsen A, Cecchin E, et al (2017) Implementing pharmacogenomics in Europe: design and implementation strategy of the Ubiquitous Pharmacogenomics Consortium. Clinical Pharmacology & Therapeutics, 101(3):341-358

^Many consortia have multiple funding sources. As a general rule in this column, only the major funders are identified. Funding from NIH and Wellcome is specifically noted, given the relevance of their policies regarding data access.

# Where no date of formation is provided, the date of the first publication is noted and the consortium is noted as being formed ‘pre’ this date.

*Where there is uncertainty as to the precise nature of the data access arrangements, inferences are drawn from website information, other documentation and funder and repository requirements (particularly NIH and dbGaP). Where a particular document is relied on to identify the data access arrangements, the title is provided in italics.
